# The Three Teachings of East Asia (TTEA) Inventory: Developing and Validating a Measure of the Interrelated Ideologies of Confucianism, Buddhism, and Taoism

**DOI:** 10.3389/fpsyg.2021.626122

**Published:** 2021-03-01

**Authors:** Yi-Ying Lin, Dena Phillips Swanson, Ronald David Rogge

**Affiliations:** ^1^Department of Counseling and Educational Leadership, The College of Saint Rose, Albany, NY, United States; ^2^Department of Human Development and Family Studies, University of North Carolina at Greensboro, Greensboro, NC, United States; ^3^Department of Psychology, University of Rochester, Rochester, NY, United States

**Keywords:** Buddhism, Taoism, Confucianism, East Asians, well-being, psychological distress, self-cultivation, mindfulness

## Abstract

**Objectives:** Buddhism, Taoism, and Confucianism have influenced societies and shaped cultures as they have spread across the span of history and ultimately across the world. However, to date, the interrelated nature of their impacts has yet to be examined largely due to the lack of a measure that comprehensively assesses their various tenets. Building on a conceptual integration of foundational texts on each ideology as well as on recent measure development work (much of which is unpublished), the current studies developed a comprehensive measure of these ideologies (the Three Teachings of East Asia Inventory; TTEA) and validated it across four languages.

**Methods:** A combined sample of 2,091 online respondents (Study 1: 322 Chinese respondents, Study 2: 400 Japanese respondents, Study 3: 362 Taiwanese respondents, Study 4: 688 White Americans and 319 Asian Americans) completed 25–35 min online survey in their preferred language: English, Traditional Mandarin, Simplified Mandarin, or Japanese.

**Results:** Exploratory Factor Analyses within a 122-item pool identified 18 stable dimensions across all samples. Measurement invariance analyses identified the final 61-items of the TTEA inventory (demonstrating reasonable invariance across all languages), confirming 18 individual tenet subscales that organize into four higher-order composites: Buddhism, Taoism, Restrictive Confucianism, and Empowering Confucianism. A shorter 36-item version of the TTEA inventory was also developed. The TTEA scales demonstrated (1) acceptable internal consistency, (2) discriminant validity, and (3) incremental predictive validity for current life satisfaction and vitality.

**Conclusions:** The TTEA inventory offers one of the first comprehensive, multilingual measures that will allow cross-cultural researchers to examine the influence of three related Eastern ideologies on societies across the world.

## Introduction

Confucianism, Buddhism, and Taoism, as philosophical ideologies, have been the three most prominent sources that scholars widely cite whenever they attempt to understand the unique worldview of East Asians. For example, these ideologies have been used to explain: (1) culturally-informed behavior (e.g., the application of Confucianism to filial piety; Hwang, 1999; Lew et al., 2011), (2) cognitive styles (e.g., differences in the use of dialectical and critical thinking; Durkin, 2008; Hamamura et al., 2008; Spencer-Rodgers and Peng, 2017), and (3) emotional processes (e.g., differences in processing and expressing emotions; Tsai et al., 2007; Murata et al., 2013). These ideologies have influenced East Asians historically, actively, and broadly, helping to shape cultural norms and influencing most aspects of East Asian societies for hundreds if not thousands of years. As these ideologies have cross-pollinated across geographical and political borders over the span of history (Lin, 1948; Gethin, 1998, p. 257; Keown, 2013, pp. 84–92; Gardner, 2014, pp. 7–15), they could likely serve as synergistic influences (enhancing one another's impacts, i.e., interdependent—see Wang et al., 2016), antagonistic influences (negating each other's impacts), and/or independent influences on cultural attitudes and behaviors. However, to determine their relative impacts would require assessing all three ideologies comprehensively and modeling them as distinct constructs within models—a task largely unaddressed in the current literature.

To date, a comprehensive measure of East Asian ideologies that would facilitate such research has yet to be developed. Although a couple self-report scales have been published to assess these ideologies [e.g., the Asian Values Scale (AVS); Kim et al., 1999; the Buddhist Coping Measure (BCOPE); Phillips et al., 2012], their strengths are balanced by some notable limitations. First, these scales were typically developed in just two languages (most commonly English and one East Asian language), prohibiting their use in cross-cultural research despite their decidedly cross-cultural focus, a striking limitation of the current measures in this area. Due to this, not only are multiple language versions unavailable, but it also remains unclear how stable the factor structures of these various scales would be across multiple cultures and languages, or whether they would demonstrate appropriate levels of measurement invariance (allowing for the direct comparisons of results across cultures). Second, some of the recent measurement work in this area has yet to undergo a full peer-review and exists only as unpublished dissertations [i.e., the Daoist thinking style questionnaire (DQ); Cott, 2012; the East Asian Relationship Norms Inventory (EARN); Park, 2009], and therefore still requires peer-reviewed validation. Third, scales of Eastern values or ideologies typically either assess an overly broad construct that fails to clearly distinguish specific ideological tenets unique to the three prominent ideologies (e.g., AVS) or take an extremely focused approach, concentrating on a single ideology (e.g., BCOPE). In part, this might be due to the complexity of that task, given the cross-pollinations of these ideologies through history and the conceptual intricacies of each ideology. Fourth, if a researcher wanted to comprehensively assess East Asian beliefs and ideologies by using the existing published and unpublished scales, it would require including (and creating new translations for) anywhere from 95 to 195 items. To advance work in this area, the current study therefore sought to develop and validate a scale [the 61-item Three Teachings of East Asia (TTEA) Inventory] across four languages and five cultural groups that comprehensively assesses key tenets of Taoism, Buddhism, and Confucianism. We would argue that the specific tenets or beliefs held by individuals across these three ideologies will serve as lenses for perceiving all aspects of life, shaping how individuals handle interpersonal and internal struggles. As a result, the development and validation of the TTEA inventory would provide researchers with a critical tool for examining specific underlying mechanisms to explain cultural differences in social norms, interpersonal and family dynamics, as well as individual coping strategies, well-being, and mental health.

### Conceptual Definitions—Defining the Key Tenets of the Ideologies

Drawing from conceptual writings on each of the ideologies (see Table 1) as well as collaborative discussions with East Asian scholars (see The Current Approach), the current study identified key tenets or beliefs uniquely associated with each of the ideologies to form our conceptual definitions and guide the development of our item pool. To begin the process of developing the conceptual foundation to ground this investigation, the first and second authors conferred with leading researchers from China, Japan, Korea, and Taiwan whose works focus on the indigenous and cultural psychology to ensure the conceptual breadth and representativeness of the tenets identified in our conceptual framework. Rather than trying to take an exhaustive approach (i.e., trying to identify and assess every possible tenet from each of the ideologies), we strove to identify a smaller set of tenets central to each ideology (i.e., highlighting the common tenets with the greatest levels of agreement from scholars in those areas). The following sections detail the results of this process for each ideology, while also placing the origins of each ideology within their larger historical contexts.

**Table 1:** Conceptual tenets operationalized for each ideology.

**Ideology** **Tenet**	**Description**	**Origin concept**	**Relevant citations**
**Buddhism**			
Not self	Releasing one's attachment to the self (not focusing on the idea of “I” or “the self”)	Anatta/Anatman (無我)	Gethin, 1998, pp. 133–162; Keown, 2013, p. 55; Thich, 2015, pp. 133–136
Karma	Active karmic view: believing in karma and using that belief to motivate positive actions	Karma (業)	Gethin, 1998, pp. 101, 129; Keown, 2013, pp. 32–33, 40–47
	Punishing karmic view: understanding negative life experiences as the inevitable repercussions of past actions		
Interconnectedness	Realizing that everything is connected and one is part of a greater whole	Pratitya Samutpada /Interdependent Co-arising (緣起)/ Idappaccayatā/Interbeing (相依性)	Shih, 1992, pp. 218–232; Gethin, 1998, pp. 74, 141–142, 155–156; Thich, 2015, pp. 221–248
Practice of meditation	Practicing meditation to quiet the mind and gain clarity in life	Meditation (定)	Keown, 2013, pp. 59, 98–101; Thich, 2015, pp. 209–210
Mindfulness	Non-judgmental awareness of one's feelings and thoughts from moment to moment	Right Mindfulness—Noble Eightfold Path (正念-八正道)	Keown, 2013, pp. 59, 103, 109–110; Thich, 2015, pp. 64–82
Impermanence	The current situation is temporary/the transitive nature	Anicca/Anitya (無常)	Gethin, 1998, p. 187; Thich, 2015, pp. 131–133; Keown, 2013, p. 55
**Taoism**			
Embracing contradiction	Embracing contradictory aspects of all things for these aspects are also complementary to one another	Taoist theory of relativity (道家相對觀)	Lin, 1948, pp. 47–55; Lao-Tzu, 1989, Ch. 2, trans.,; Lao-Tzu, 1997, Fu, 2018b
Non-interference	Realizing that sometimes the best course of action is to refrain from taking intentional action	Wu-Wei (無為)	Lin, 1948, pp. 68–69, 194–197, 229–230; Henricks, 1989, xxi; Lao-Tzu, 1989, Chs. 2, 37, 48, trans.,; Lao-Tzu, 1997, trans.
Zi-Ran	Accepting all things as they are	Zi-Ran (自然)	Lin, 1948, pp. 125–127; Lao-Tzu, 1989, Chs. 17, 18, 19, 64, trans.; Lao-Tzu, 1997, trans.
Cyclic nature	Understanding life is a cycle which leads us through peaks and valleys	Cyclic nature (天道循環)	Lin, 1948, pp. 145–149, 207–208; Lao-Tzu, 1989, Chs. 25, 40, trans.; Lao-Tzu, 1997, trans
Tranquility	Remaining calm and still throughout daily life	Tranquility (寧靜)	Lin, 1948, pp. 106–113; Lao-Tzu, 1989, Chs. 15, 16, trans.; Lao-Tzu, 1997, trans.
**Confucianism**			
Propriety	Propriety Pressure: a need to uphold appropriate behavior and action in public because of social pressure	Propriety (禮/Li)	Goldin, 2011, pp. 19–23; Hwang, 2012, pp. 108–109, 114–116; Gardner, 2014, pp. 25–28
	Intrinsic Propriety: presenting proper actions voluntarily regardless surrounding environments		
Interpersonal harmony	Avoiding conflicts by withholding negative feelings or being agreeable with others	Interpersonal harmony (和諧)	Ho, 1995; Hwang, 2012, pp. 126–128, 338–340
Conforming to social norms	Recognizing and adhering to social norms and expectations.	Yi as socially appropriate norms (義—宜也)	Hwang, 2012, pp. 108–109; Tan, 2014; Fu, 2018a, pp. 160, 341–344
Relational hierarchy	Respecting and valuing perspectives of elders and/or authorities	Yi as socially appropriate norms (義—宜也)	Hwang, 2012, pp. 108–109; Tan, 2014; Fu, 2018a, pp. 71–83, 160, 341–344
Self-cultivation	Cultivating the best of the self	Self-cultivation (修養)	Goldin, 2011, p. 5; Hwang, 2012, pp. 116–121; Gardner, 2014, pp. 16–18
Leading by example	Modeling one's desired behavior to inspire others	Self-cultivation (修養) & Jun-Zi (君子)	Goldin, 2011, pp. 5, 117; Hwang, 2012, pp. 121–125; Gardner, 2014, pp. 16–18
Human heartedness	Trying to be kind, loving, and loyal to the people in my life	Ren (仁)	Goldin, 2011, p. 18; Hwang, 2012, pp. 121–122; Gardner, 2014, p. 22–25

#### Confucianism

Founded by Confucius (孔子, 551–479 B.C.), Confucianism aims to create a social and moral structure by defining virtues and preferred methods of navigating interpersonal relationships. The structure is grounded in three distinct yet related main principles (Huang and Charter, 1996; Hwang, 2012, p. 108): *benevolence* (仁/ren, i.e., loving all human beings), *righteousness* (義/yi, i.e., making decisions based on justice, ethics, and altruism), and *propriety* (禮/li, i.e., behaving in socially proper manners). Benevolence is considered the core principle and the other two principles can be understood as means to help individuals put benevolence into action to navigate social situations (Hwang, 2012, p. 109), thereby defining preferred virtues and basic social etiquette. Confucianism therefore emphasizes the importance of maintaining interpersonal harmony and relational hierarchy, promoting a society dominated by relationships and established social roles (Ho, 1995; Hwang, 2012, pp. 160–161).

Based on this conceptual structure, eight tenets were drawn from Confucianist writings to represent key facets of Confucianism (see Table 1). We conceptualized benevolence as a combination of the tenets of *Self-Cultivation* (i.e., cultivating the best of the self), *Leading by Example* (i.e., modeling one's desired behaviors to inspire others), *Human Heartedness* (i.e., trying to be kind and loving the people in one's life). We conceptualized propriety as a combination of *Propriety Pressure* (i.e., a need to uphold appropriate behavior and action in public) and *Intrinsic Propriety* (i.e., presenting proper actions voluntarily regardless surrounding environments). To represent righteousness, we identified the tenet of *Conforming to Social Norms* (i.e., recognizing and adhering to social norms and expectations) as a form of that principle. Finally, the application of those three main principles to interpersonal relationships was measured by *Relational Hierarchy* (i.e., respecting, valuing, and deferring to the perspectives of elders and/or authorities) and *Interpersonal Harmony* (i.e., avoiding conflicts by suppressing one's opinions or negative feelings to prioritize being agreeable with others). As exemplified by these tenets, many of the tenets of Confucianism are focused on restricting individual needs and desires for the greater good of the larger community (e.g., propriety pressure, conforming to social norms, relational hierarchy), and these are balanced by a set of tenets that encourage individuals to strive toward becoming the best possible version of themselves (e.g., self-cultivation, leading by example, human heartedness) to further improve society.

#### Taoism

Taoism, originating from the same period of Chinese history as Confucianism, offers a distinctive worldview in comparison to Confucianism. Lao-Tzu (date unknown), a mysterious figure, was credited with the establishment of Taoism and offered the insight of the Tao to Confucius (Fu, 2018b; Minford, 2018). The teachings of Taoism center on the concepts of “Tao.” In Chinese, Tao literally means “the way.” Based on Tao-Te Jing, although “the way” can be partially explained in texts, it can only be fully realized by shaping one's own behavior to follow the laws of Nature (Chan, 2018). The use of the term Nature (自然/Ziran) here does not simply refer to the natural world but rather a fundamental stance toward life marked by acceptance, surrender, and tranquility to promote inner peace marked by the states of the “unconditioned” and “so-of-itself” (see Lao-Tzu, 1997, 2018, Chapter 25 for a review). The laws of *Nature* are manifested in nature's constant changes and its cyclic, circular constitution (e.g., four seasons, ebb and flow; Hwang, 2012, p. 103). Therefore, people practicing Tao would not force their way through life, instead they would instead strive to enjoy the state of tranquility, embrace internal wisdom, let go of control, and harmonize their life with Tao (see Minford, 2018 for a review).

Drawing on this conceptualization, the current study focused on five widely accepted tenets to capture core features of Taoism (see Table 1). We conceptualized the spirit of following the laws of *Nature* as a combination of the tenets of *Cyclic Nature* (i.e., understanding life is a cycle which leads us through peaks and valleys) and *Embracing Contradiction* (i.e., embracing contradictory aspects of all things for these aspects are also complementary to one another). We conceptualized the state of forgoing control and harmonizing with the world as the tenet of *Zi-Ran* (i.e., accepting all things as they are). Finally, we conceptualized the strategies of not forcing one's way through life and maintaining inner peace as a combination of the tenets of *Non-interference/Wu-Wei* (i.e., realizing that sometimes the best course of action is to refrain from taking intentional action) and *Tranquility* (i.e., remaining calm and still throughout daily life), respectively. Thus, as exemplified by these tenets, Taoism seeks to help individuals align with fundamental laws of nature, approaching life in a more accepting, peaceful, tolerant, and tranquil manner.

#### Buddhism

Founded by Gautama in India (roughly around 500–400 B.C.; Keown, 2013, p. 17), Buddhism began to infiltrate China around the first century A.D., spreading to Korea in the fourth century, and to Japan in the sixth century (Chen, 2006). Despite evolving into several distinct branches, Buddhism is unified by one common goal—attaining enlightenment to reach Nirvana through the process of “awakening.” Toward that end, Buddhist teachings encourage individuals to understand the law of cause and effect (i.e., Karma), to discard the tendency to cling to one's sense of self (Bhikkhu, 1996, 2013), to realize the existence of everything depends on the existence of another (Shih, 1992, pp. 218–232; Thich, 2015, pp. 221–248), and to know that most things change (Keown, 2013, p. 55–56). Buddhism encompasses a variety of traditions (most commonly meditation) in order to achieve a state of non-judgmental attentive awareness to clearly see the roots of suffering and gain inner wisdom (e.g., Thich, 2015).

Based on this conceptualization, we identified seven key tenets of Buddhism from a range of Buddhist writings (see Table 1). We conceptualized individuals' understanding of the law of cause and effect with the tenets of karma. Based on recent work (Phillips et al., 2012) we identified two distinct aspects of karma: an *Active Karmic View* (i.e., believing in karma and using that belief to motivate positive actions) and a *Punishing Karmic View* (i.e., understanding negative life experiences as the inevitable repercussions of past actions). We conceptualized the discarding of one's tendencies to cling to the self as the tenet of *Not-Self/Anatta* (i.e., releasing the attachment to the self). This tenet draws upon Buddha's suggestion that the process of mentally distinguishing the “self” from “others” will cause individuals to experience suffering as it engenders an emotional attachment and clinging to that self (e.g., Thich, 2015). We conceptualized individuals' understanding of how everything is related to one another with the tenet of *Interconnectedness* (i.e., realizing that everything is connected and one is part of greater whole). We conceptualized the knowledge of transient nature of everything with the tenet of *Impermanence* (i.e., the current situation is temporary). Finally, we conceptualized the tenets of *Meditation* (i.e., practicing meditation to quiet the mind and gain clarity in life) and *Mindfulness* (i.e., non-judgmental awareness of one's feelings and thoughts from moment to moment) as key components of the daily practice of Buddhism. Thus, although somewhat similar to Taoism, Buddhism (as exemplified by the tenets above) encourages the cultivation of a non-judgmental, open, and accepting attentive awareness to the present moment (grounded in the practice of meditation) that allows individuals to decenter from suffering (i.e., dukkha), holding the power to ultimately transform that suffering into liberation and enlightenment (Bodhi, 1994).

#### Contextualizing Domains of Impact

We believe that Taoism, Buddhism, and Confucianism are not only distinct at the conceptual and metaphysical level but are also applied to daily life in widely different ways, particularly for individuals living in East Asia. For instance, Confucianism mostly governs the *structure of social system and moral requirement*s standards (Ho, 1994, 1995) and has been a source for socialization and external standards (Ho, 1989), potentially shaping how individuals perceive morality, their own roles in the larger social structure, and appropriate behaviors across different contexts (Fu, 2018a). In contrast, the teachings of Buddhism and Taoism primarily offer individuals *sources of coping and support* (Lin, 1948; Zhang et al., 2002; Tweed et al., 2004; Chen, 2006), more directly impacting how they navigate the stress and demands of daily life. Thus, we anticipate differing patterns of association between the various ideologies (as well as their tenets) and aspects of daily life like emotional coping, relationship expectations, life satisfaction, and psychological distress.

#### Worldwide Dissemination

Although each of these ideologies originated in a very localized manner, as each of these ideologies grew and spread across history, they infiltrated East Asia to varying degrees, and now transcend regional and national borders, often developing regional variations as they were integrated into local cultures. Thus, an individual living within a specific town or city within East Asia could have been acculturated to the tenets of all three major ideologies to varying degrees. In fact, as the writings of these ideologies have been translated into an increasing diversity of languages, we would argue that the tenets of these ideologies have even spread worldwide, gaining relevance across numerous cultures. For example, growing out of Buddhist writings, the last 25 years has seen an explosion of research on Mindfulness and Mindfulness-based interventions that use meditation to help alleviate suffering within samples drawn predominantly from Western countries (e.g., Kabat-Zinn, 2003), highlighting the salience of mindfulness within the daily lives of all individuals. Similarly, Tao-Te Ching as one of the most translated book in the world (Minford, 2018) and self-help books like “*The Tao of Pooh*” (Hoff et al., 1982) served to spread and popularize the tenets of Taoism in western cultures. Given the reverberations of these ideologies across the world, we posited that a measure assessing the tenets of Buddhism, Taoism, and Confucianism would have relevance (i.e., predictive validity) in the lives of individuals in all countries, providing a rich method of assessing and conceptualizing differences between individuals within the same country and/or culture, as well as differences between various countries/cultures.

### Previous Approaches to Measuring East-Asian Ideologies

Some of the most widely used scales conceptualize East Asian values as a single global-dimension, sacrificing conceptual precision to create a single dimension scale targeting the largest differences between Western and East Asian ideologies (which usually highlights the presence of a Confucian worldview). For example, the Asian Values Scale (AVS; Kim et al., 1999) is a 36-item scale (currently validated in English, traditional Mandarin, simplified Mandarin, Korean, and Japanese) that yields a single score representing the degree to which individuals embrace the more restrictive aspects of Confucianism: conforming to social norms and expectations and subjugating their own needs and emotions to prioritize the needs of the larger social group. Alternatively, previous scales have focused on anywhere from one to a small handful of very specific tenets instead of capturing the broader meta-constructs of Confucian, Buddhist, or Taoist ideologies. For example, Peng and Nisbett (1999) and Spencer-Rodgers et al. (2004, 2010) examined dialectic thinking and dialectic self as key tenets to represent a Taoist worldview using (as of yet) unpublished scales (i.e., lacking peer-reviewed measurement papers focused on their validation and psychometric properties). Similarly, Wang and colleagues (2016) utilized single tenets from each of these ideologies [i.e., interpersonal harmony from Confucianism (in English and Traditional Mandarin), dialectic coping from Taoism (a three-item scale in English in Traditional Mandarin), and non-attachment from Buddhism (in Traditional Mandarin)] to examine their benefits for mental health. Finally, some scholars conceptualized common mechanisms that might be shared across Confucianism, Buddhism, and Taoism (e.g., collectivism, Triandis, 1995; interdependent self-construal, Markus and Kitayama, 1991) without distinguishing the peculiar contributions of those different East Asian ideologies. Although an effective strategy, focusing on constructs like collectivism runs the risk of over-simplifying the true complexity of the influences these tenets on the lives of individuals and thereby over-simplifying cross-cultural differences between Eastern and Western countries. Aside from the handful of studies just reviewed, focused measure development work in the area of Eastern ideologies has been somewhat scant, thereby limiting the abilities of researchers to explore the cross-cultural impact of these ideologies on the daily lives of individuals. Although a couple of the previous scales have been translated into a wider number of languages, many of the scales were developed in just one or two languages (e.g., English and traditional or simplified Mandarin), further restricting their cross-cultural use. In addition, the measurement work in assessing East Asian ideologies has been somewhat haphazard and piecemeal, lacking a comprehensive framework to integrate those efforts (and the various constructs they assess) and clearly link them to their conceptual origins within Buddhism, Taoism, and Confucianism.

### The Current Approach

#### A Cross-Cultural Strategy

We would assert that a comprehensive scale that precisely assesses these three interrelated ideologies *via* their unique sets of tenets is critical to fully appreciate subtle but important within-group differences across the heterogeneous ideological landscape of East Asia as well as the degree to which these various ideologies have penetrated the Western world. We therefore collected distinct samples from a variety of countries/cultural backgrounds (China, Taiwan, Japan, and United States—both White and Asian American subjects) across the key languages representing those populations (simplified Mandarin Chinese, traditional Mandarin Chinese, Japanese, and English). This ensured that the resulting scale would be validated and immediately available for cross-cultural research.

#### Blending Conceptual With Data-Driven Methods

Drawing from existing published and unpublished measures and developing additional items when necessary (see Supplementary Table 1), we curated a pool of 122 items that aligned with the 20 tenets specified within our conceptual definitions. We chose to identify the final items and validate the TTEA inventory in a data-driven and exploratory manner. Thus, instead of using a panel of experts to initially trim the curated item pool, we decided to collect responses to the full item pool across all five samples of subjects, allowing us to run analyses to identify: (1) the items most effectively and consistently assessing each tenet across all languages, and (2) the higher-order organization of the tenets into discrete ideologies. Our work was therefore heavily informed by: (1) conceptual and theoretical work in this area, (2) previous (largely unpublished) measurement work in this area, and (3) how individual items and tenets performed within the analyses run in the current large and diverse sample spanning five culture groups across four languages.

#### Empowering Individual Tenets

We purposefully grounded our definitions of the three main ideologies by identifying specific tenets to be assessed. In part, this strategy allowed us to embrace the complexity of those ideologies and their associated teachings. However, this also allowed for the creation of a scale that could be sensitive to individual differences. Thus, even if an individual was raised and acculturated in a strongly Confucianist environment/society, they might have internalized certain tenets (e.g., interpersonal harmony—avoiding conflict with others by suppressing one's feelings) to a greater or lesser extent than other tenets (e.g., human heartedness—striving to be kind and loving to others). Furthermore, even for individuals that have strongly internalized most of the tenets of a specific ideology like Confucianism, individual tenets might show stronger or weaker links within specific interpersonal relationships (e.g., friends vs. coworkers), to specific dynamics (e.g., expressing support vs. expressing disagreement or hurt), and to individual distress and well-being (e.g., Wang et al., 2016). For example, the Confucianism tenets of interpersonal harmony (avoiding conflict with others by suppressing one's feelings) and human heartedness (striving to be kind and loving to others) are both drawn from the same ideology. However, it is quite possible they could have different impacts, with the restrictive tenet of interpersonal harmony being associated with lower well-being and the empowering tenet of human heartedness being associated with greater well-being. Thus, by creating a scale comprehensively assessing as many as 20 distinct subscales, the TTEA inventory would offer researchers with up to 20 unique constructs to help model the complexity of cultural differences in cross-cultural studies.

#### Establishing Links to Social Behaviors and Individual Well-Being

As scales assessing Buddhism, Taoism, and Confucianism are only just beginning to emerge within the literature, the exact nature of the links between these ideologies and individual functioning or individual well-being remain less clear. This is particularly true for the individual tenets of these various ideologies. However, given the rigid social constraints associated with Confucianism, we anticipated that endorsement of the more restrictive and self-denying tenets of Confucianism would be associated with higher levels of collectivism, filial piety, and face concerns and with lower levels of well-being. As Buddhist teaching promote the practice of decentering from suffering, we anticipated that endorsement of Buddhist beliefs might be associated with higher levels of well-being (e.g., greater peace of mind, life satisfaction, and the ability to be non-reactive to difficult experiences). Similarly, as Taoist teachings emphasize embracing the cyclic nature of life, endorsement of Taoist beliefs might also be associated with greater peace of mind, life satisfaction, and non-reactivity.

#### The Current Studies

To accomplish the goals just outlined, an item pool of 122 items was created in English from 71 items drawn from eight existing measures and 51 items developed by the authors to enrich and diversify the conceptual content of the pool—ensuring that each proposed tenet was represented by multiple items. That pool of items (along with all study materials) was translated (and back-translated) into Japanese, traditional Mandarin, and simplified Mandarin. Four studies were then conducted—one in each language—yielding five culturally distinct samples: 322 people from China, 400 people from Japan, 362 people from Taiwan, 319 Asian Americans, and 688 White Americans. We then used exploratory and confirmatory factor analyses, along with correlational analyses and measurement invariance analyses to develop and validate the 61-item Three Teachings of East Asia (TTEA) Inventory across those cultural groups.

## Methods

### Participants

As shown in the top half of Table 2, the sample was made up of individuals primarily in their 20's, 30's, and 40's who were mostly female (69%), fairly educated (65% with a college degree), working full-time jobs (57%), and in romantic relationships (60%). For Asian Americans, 265 (83.1%) identified as people of East Asian descents (i.e., Chinese, Japanese, Korean, and Taiwanese). 12.2% were foreign-born first-generation immigrants and 76.2% were second generation individuals who born and raised in the US. Although significant differences emerged across the five cultural groups examined, those differences were fairly modest. Almost a third of the respondents (31%) identified as atheist, 23% as Christian, 22% as Buddhist, 7% as Taoist, and 1.5% Shinto (a blend of indigenous beliefs and Buddhism within Japan Cartwright, 2017). The demographics presented across the samples suggest that the studies were successful in recruiting a diverse array of individuals from each of those countries.

**Table 2:** Demographics of the cultural subsamples and tests for differences.

	**Overall**	**White American**	**Asian American**	**China**	**Japan**	**Taiwan**	**χ^**2**^ or ANOVA omnibus test of differences**
**Sample size**	2,091	688	319	322	400	362	
**Demographic indices**							
Female	69%	81%^A^	60%^CD^	48%^D^	71%^B^	67%^EC^	${\text{χ}}_{\left(4\right)}^{2}$ = 130.5^*^
Age: Mean(SD)	32.42 (10.22)	31.5^B^(10.04)	29.9^B^ (9.81)	27.1^C^(6.29)	37.1^A^ (6.29)	35.9^A^(10.80)	*F*_(4, 2, 086)_ = 67.0^*^
Income^1^: Mean(SD)	$39,000 (32,040)	$53,429^A^(31,915)	$55,601^A^ (31,762)	$22,931^C^(25,553)	$41,183^B^ (27,296)	$37,624^B^(31,226)	*F*_(4,2,066)_ = 72.8^*^
Bachelor's degree or higher	65%	73%^A^	66%^AB^	62%^B^	44%^C^	77%^A^	${\text{χ}}_{\left(4\right)}^{2}$ = 117.1^*^
Full-Time Student	16%	18%^A^	23%^A^	20%^A^	6%^B^	17%^A^	${\text{χ}}_{\left(4\right)}^{2}$ = 43.9^*^
Part-Time Student	8.6%	11.4%^A^	10.8%^AB^	8.5%^AB^	5.4%^B^	5.1%^B^	${\text{χ}}_{\left(4\right)}^{2}$ = 19.5^*^
Full-Time Work	57%	64%^AB^	58%^B^	67%^AB^	31%^C^	71%^A^	${\text{χ}}_{\left(4\right)}^{2}$ = 160.5^*^
Part-Time Work	19%	19%^AB^	27%^A^	14%^EC^	26%^A^	10%^C^	${\text{χ}}_{\left(4\right)}^{2}$ = 51.0^*^
In Romantic Relationships	60%	69%^A^	60%^A^	63%^A^	42%^B^	59%^A^	${\text{χ}}_{\left(4\right)}^{2}$ = 75.3^*^
Married	39%	33%^EC^	29%^C^	36%^EC^	55%^A^	41%^B^	${\text{χ}}_{\left(4\right)}^{2}$ = 67.6^*^
Had Children	31%	30%^EC^	26%^C^	28%^CB^	73%^A^	39%^EC^	${\text{χ}}_{\left(4\right)}^{2}$ = 166.6^*^
Currently in Counseling	11.5%	21%^A^	8.6%^EC^	3.4%^C^	11%^B^	4.2%^C^	${\text{χ}}_{\left(8\right)}^{2}$ = 119.4^*^
**Self-identified religion/beliefs**							
Christianity	23%	45%^A^	34%^A^	6.2%^B^	4.3%^B^	8%^B^	${\text{χ}}_{\left(4\right)}^{2}$ = 392.2^*^
Jewish	1.4%	3.3%	1.3%	0.0%	0.5%	0.0%	see note 2
Buddhism	22%	2.5%^C^	20%^B^	25%^B^	45%^A^	30%^B^	${\text{χ}}_{\left(4\right)}^{2}$ = 299.2^*^
Philosophical Taoism^2^	3.2%	0.6%	1.6%	5.3%	0.0%	11%	–
Religious Taoism^2^	3.6%	0.0%	1.6%	2.2%	0.5%	17%	–
Shinto^2^	1.5%	0.0%	0.0%	0.0%	8%	0.0%	–
Religious Naturalism/Shamanism	1.1%	1.3%	1.3%	1.6%	1.0%	0.6%	${\text{χ}}_{\left(4\right)}^{2}$ = 1.86
Atheism	31.4%	24%^B^	23%^EC^	55%^A^	37%^B^	27%^B^	${\text{χ}}_{\left(4\right)}^{2}$ = 115.6^*^
Other	12.9%	23%^A^	17%^A^	5.3%^B^	4.0%^B^	7%^B^	${\text{χ}}_{\left(4\right)}^{2}$ = 122.0^*^

1 *All incomes were converted to US dollar equivalents*.

2 *χ^2^Could not be calculated for variables with empty cells (i.e., religions with 0.0% prevalence rates in at least one cultural group). Given the number of contrasts presented in this table, we used a p < 0.001 threshold to determine significance to help constrain the experiment-wide Type I error rate*.

* *Thus, an indicates p < 0.001. Any omnibus test across the five groups revealing significant differences was followed up by pairwise comparisons to determine where the differences existed. Superscripted letters following statistics indicate the results of these pairwise comparisons across the groups represented in a row with different letters indicating significantly different values between groups on that variable*.

### Procedure

The studies and all of their associated materials were evaluated and approved by a university institutional review board to ensure adherence to ethical standards for human subjects research. The survey was presented online using the surveygizmo platform and the first page of the survey allowed respondents to select the language in which the survey would be presented. The respondents were then presented with information sheets in their chosen language to obtain informed consent prior to providing any survey responses. Participants has to be at least 18 years of age to participate (to ensure they could provide their own consent).

#### Recruitment

Respondents were recruited entirely online, primarily using the title of the study (“*The Tenets of Life Study*”) and brief language explaining that: (1) participation was voluntary, (2) online, (3) involved a 25–35 min survey, and (4) could be discontinued at any time. Much of the recruitment was conducted with crowdsourcing platforms like Amazon.com's Mechanical Turk service, and respondents from those systems received $0.40–$0.60 cents (in the currency of their country of residence) as incentives for participating.

#### Study 1 (United States) Recruitment

The sample of individuals living in the US was recruited from Amazon.com's Mechanical Turk service (a crowdsourcing platform; 45.9%), ResearchMatch (48.8%), and other methods (e.g., university email systems, listserves, facebook; 5.3%).

#### Study 2 (China) Recruitment

The sample of individuals living in China and/or self-identified as Chinese was primarily recruited from Witmart (85.1%), a crowdsourcing platform popular in China, Mechanical Turk (10.0%) and other methods (e.g., email, listserves; 4.9%).

#### Study 3 (Japan) Recruitment

The sample of individuals living in Japan and/or self-identify as Japanese was recruited primarily from Crowdworks (95.5%), a crowdsourcing platform popular in that country and from Mechanical Turk (4.5%).

#### Study 4 (Taiwan) Recruitment

As an equivalent crowdsourcing platform was not available in Taiwan, the vast majority of individuals living in Taiwan or identifying as Taiwanese (72.9%) were recruited by contracting with a professional survey service (Pollster). A smaller proportion (27.1%) were recruited by other methods (e.g., emails, facebook, and listserves).

#### Translation Process

The entire survey including the information sheet and the recruitment materials were translated from English to simplified Mandarin, traditional Mandarin, and Japanese. All translators were fluent in English and their relevant languages and had at least 2 years of translation experience. For the Japanese version, the initial English-to-Japanese translation and the Japanese-to-English back translation were conducted by two separate professional translators. Discrepancies were resolved *via* discussion between the first author and the first translator. For both Mandarin translations, the first author was primarily responsible for the English-to-Chinese translation and was assisted by two doctoral students from China and Taiwan, studying in related fields to hone the translations. A professional translator then was used for back-translation. Discrepancies were resolved by consensus among the first author and the two doctoral students.

### Measures

Unless otherwise specified, the items of all scales were presented on common six-point Likert scales (“*Never*” to “*Always*”). Answers were averaged so that higher scores reflect higher levels of the construct being assessed. Cronbach alphas were estimated in each cultural group.

#### Item Pool

A pool of 122 items assessing tenets of East Asian ideologies (51 items assessing tenets of Confucianism, 38 assessing tenets of Buddhism, and 33 assessing tenets of Taoism) was curated for the current study (see Supplementary Table 1). To build on previous published and unpublished measurement work in this area, a total of 71 items in that pool were drawn from seven existing measures: the East Asian Relational Norms Inventory (EARN; Park, 2009; Park et al., 2011; not yet published in a peer-reviewed journal), the Asian Values Scale (AVS; Kim et al., 1999), the Asian American Values Scale-Multidimensional (AAVS-M; Kim et al., 2005), the Buddhist Coping Measure (BCOPE; Phillips et al., 2012), the Multidimensional Psychological Flexibility Inventory (MPFI; Rolffs et al., 2016), the Dialectical Self Scale (DSS; Spencer-Rodgers et al., 2015), and the Daoist Questionnaire (DQ; Cott, 2012; not yet published in a peer-reviewed journal). Based on the conceptual definitions of the targeted tenets for each of the three ideologies, the authors wrote another 51 items to augment the item pool, diversifying the pool beyond the content of the previously developed published and unpublished scales. This included additional items to increase and deepen the representation of specific tenets within the pool as well as generating entirely new sets of items assessing tenets unassessed by previous scales (i.e., self-cultivation, well-defined roles, benevolence, unspeakable wisdom, and cyclic nature). These items were written in collaboration by the three authors and were written to maximize their alignment with the language used to describe each of the tenets within classic texts on Buddhism (e.g., Bhikkhu, 2013; Thich, 2015), Taoism (e.g., Lao-Tzu, 1948; Lin, 1948), and Confucianism (e.g., Hwang, 2012; Gardner, 2014). As mentioned above, in lieu of subjecting the items to an initial qualitative review by a panel of subject experts and trimming the item pool based on those subjective opinions, we chose instead to make use of a panel of experts in developing our conceptual definitions (at a key earlier stage of the development process). We then chose to retain the full diversity of the 122-item pool and use exclusively quantitative findings from a markedly large and diverse sample spanning five cultures and four languages to select the final items for the TTEA scale. In this manner, we balanced both developing a comprehensive conceptual framework as a foundation for the measure with prioritizing a rigorous empirical development and validation of the resulting scale to ensure its robust psychometric properties across all languages and cultural groups.

#### Culturally Informed Behavior Measures

*Collectivism* was assessed with the three positively worded items of collectivism subscale of the Asian American Values Scale—Multidimensional (AVSM, Kim et al., 2005), which demonstrated appropriate internal consistency across the five cultural groups and four languages of the current study (α's ranging from 0.88 to 0.94 across the cultural groups). *Dual Filial Piety* was assessed with the eight-item reciprocal subscale (α's ranging from 0.91 to 0.94) and the eight-item authoritarian subscale (α's ranging from 0.80 to 0.92) of the Dual Filial Piety scale (DFPS; Yeh and Bedford, 2003). *Face Management* was assessed with four items of the Face Concerns Scale (FCS; Ting-Toomey and Oetzel, 2001; Oetzel and Ting-Toomey, 2003; α's ranging from 0.88 to 0.91) focused on saving face for one's self (e.g., “*I am concerned with protecting my self-image*”), and with four items of the Loss of Face scale (LOF; Zane and Yeh, 2002; α's ranging from 0.85 to 0.90) focused on saving face for others (e.g., “*When discussing a problem, I make an effort to let the person know that I am not blaming him or her*”).

#### Individual Functioning

The survey included a number of measures of individual functioning. *Embarrassment toward counseling* was assessed with a single item, “*I would be deeply embarrassed getting professional help for emotional problems*.” *Positive attitudes toward counseling* were assessed with three items (“*I believe that anyone could benefit from professional help for emotional problems at tough points in their lives*,” “*If you were struggling with emotional problems, how likely is it that you would seek professional help*,” “*If someone close to you was struggling with emotional problems, how likely is it that you would encourage them to seek professional help*,” α's ranging from 0.72 to 0.84). *Mindfulness* (e.g., “*I was in tune with my thoughts and feelings from moment to moment*”) was assessed with five-item present moment awareness subscale of the Multidimensional Psychological Flexibility Inventory (MPFI; Rolffs et al., 2016; α's ranging from 0.90 to 0.94). *Inattentive/unawareness* (e.g., “*I did most things on ‘automatic' with little awareness of what I was doing*”) was assessed with the five-item lack of present moment awareness subscale of the MPFI (α's ranging from 0.90 to 0.96). *Non-reactivity/defusion* (e.g., “*I was able to let negative feelings come and go without getting caught up in them*”) was assessed with the five-item cognitive defusion subscale of the MPFI (α's ranging from 0.90 to 0.94). *Judging thoughts and feelings* (e.g., “*I thought some of my emotions were bad or inappropriate and I shouldn't feel them*”) was assessed with the five-item self-as-content subscale of the MPFI (α's ranging from 0.84 to 0.95). *Peace of Mind* (e.g., “*I had peace and harmony in my mind*,” “*My mind was free and at ease*”) was assessed with the five positively worded items of the Peace of Mind scale (POM; Lee et al., 2013; α's ranging from 0.95 to 0.96). *Life satisfaction* was assessed with the five-item Satisfaction with Life Scale (SWLS; Diener et al., 1985; α's ranging from 0.88 to 0.94). *Psychological distress* (e.g., “*In the last month… I felt depressed, I felt discouraged*”) was assessed with six items strongly loading onto the corresponding factor of the Mood and Anxiety Symptom Questionnaire (MASQ; Watson et al., 1995a,b; α's ranging from 0.95 to 0.96). *Somatic anxiety* (e.g., “*In the last month… I felt dizzy or light-headed, I was trembling or shaking, My hands were cold or sweaty*”) was assessed with six items strongly loading onto the corresponding factor of the MASQ (α's ranging from 0.90 to 0.94).

## Results

### Developing the TTEA Inventory

#### Item Selection

To identify the final 61 items of the TTEA inventory, we ran a *preliminary* Exploratory Factor Analysis (EFA, using SPSS 23.0) on the larger item pool of 122 items within the full sample, using principle axis factoring with Direct Oblimin rotation to allow the factors to correlate. The Kaiser-Guttman criteria identified 20 factors with eigenvalues >1.0, yielding factors that accounted for 69.7% of the variance and mapped directly onto the 20 anticipated tenets. When we tried to extract 21 factors (to explore the stability of this EFA solution), the additional factor failed to contain a single item (i.e., no items with loadings ≥0.40 and no items with their strongest loadings on that factor), further supporting a 20-factor solution. We then conducted similar EFA analyses within each of the culture groups. Taken as a set, these item-pool trimming EFAs allowed us to identify 61 items comprising 18 factors that: (1) consistently emerged across all cultural groups, (2) conceptually aligned with the expected tenets, and (3) contained the 2–4 items most internally consistent (i.e., highest factor loadings—typically ≥0.50) for each subscale. This also allowed us to screen out items that failed to robustly load on any of the main factors (i.e., loading <0.40 on all of the factors). A final set of 61 items were therefore chosen for the TTEA inventory. Two additional factors consistently emerged from the EFAs in the five cultural groups: impermanence and mindfulness. Although these factors mapped onto targeted tenets within our conceptual definitions, EFAs examining the higher order structure of the factors extracted (see below) suggested that those two tenets demonstrated strong cross-loading across the three main East Asian ideologies (i.e., mindfulness correlated 0.48 with Buddhism, 0.54 with Taoism, and 0.51 with Empowering Confucianism; impermanence correlated 0.41 with Buddhism, 0.50 with Taoism, and 0.52 with Empowering Confucianism). Thus, those two tenets were evenly associated with Taoism, Buddhism, and Confucianism, suggesting that they might be common and pervasive aspects of these three Eastern ideologies in general. As one of the goals of the TTEA inventory was to distinguish among those three major ideologies, we chose not to include those subscales on the final measure.

#### Shortened Version

Although the full 61-item version of the TTEA inventory will provide researchers with a comprehensive tool for evaluating and deconstructing cultural differences (providing four composite scores and 18 individual tenet scores to be examined as possible mechanisms), the length of the scale might hinder its adoption across a wide range of future studies. Thus, after selecting the final 61 items of the TTEA inventory, the two most prototypical (i.e., conceptual definition consistent) items of each subscale were selected to create a shorter 36-item version of the scale.

#### Measurement Invariance Across Cultural Groups

To determine if the final items of the TTEA inventory functioned comparably across all five cultural groups, we used the multi-stage process outlined by Van de Schoot et al. (2012) involving a series of Confirmatory Factor Analyses (CFA; run in Mplus 7.11) on a model specifying 61 items loading on 18 correlated subscales (focusing on item-level MI) to evaluate measurement invariance. We specifically used the parameterization setting the means and variances of the latent subscale factors to 0 and 1, respectively, to focus the analyses on the equivalence of the factor loadings (see Figure 2A in Van de Schoot et al., 2012). *Step 0—Configural Invariance (i.e., weak factorial invariance)*. As shown in the upper portion of Table 3, when that model was estimated in a multiple-group analysis with no constraints between the groups (i.e., allowing the solution to freely vary across the groups), it demonstrated adequate fit. When that model was estimated in each of the culture groups separately, the models continued to demonstrate adequate fit, indicating that the CFA model is valid in each of the groups and suggesting a common factor structure across all five groups and all four language versions of the TTEA inventory. *Step 1—Metric Invariance (i.e., strong factorial invariance)*. When the factor loadings of the model were constrained to be equal across the five culture groups, the multiple-group model continued to demonstrate reasonable fit (see Table 3), suggesting that respondents attributed the same meaning to the latent dimensions of each tenet across the different translations and groups. *Step 2—Intercept Only Invariance*. When the intercepts for the latent tenet dimensions were constrained to be equal across the culture groups, the model demonstrated slightly poorer fit as the TLI fell just below the 0.90 cutoff (TLI = 0.894). Following the guidelines outlined by Van de Schoot et al. (2012), we then released individual items of the scale within specific groups maintaining the constraint that only one or two items could be released from each subscale within each group (ensuring that the majority of the items on the subscale were still constrained across groups). By releasing roughly 1 out of every 9 or 10 items on the scale, the model demonstrated acceptable fit. This partial invariance suggests that the meanings of the levels of a majority of the items remained reasonably equivalent across groups (i.e., there were roughly equivalent mean levels of responding to the various translations of each item). *Step 3—Partial Scalar Invariance (i.e., strict factorial invariance)*. When both the factor loadings and (a large majority of) the intercepts were constrained to be equal across the groups, the model continued to demonstrate adequate fit (Table 3), suggesting sufficient measure invariance to support scores being directly compared across groups on the TTEA subscales (see Van de Schoot et al., 2012). *Step 4—Full Uniqueness Measurement Invariance*. Finally, when the model from step 3 was also constrained to have equal item residuals across groups, the model continued to demonstrate adequate fit, suggesting that the 18 tenets are essentially measured reasonably identically across all five groups and four languages, at least for the majority of items on the scale. Taken as a set, these results support the cross-cultural measure invariance of the versions of the TTEA inventory developed in this study. Given this invariance, the remaining CFA models were conducted in the full sample.

**Table 3:** Confirmatory factor analyses examining measurement invariance and correlational structure of the TTEA.

**Stage of analysis**	**χ^**2**^**	**# of param**	**CFI**	**TLI**	**RMSEA**	**90% C.I**.		**AIC**	**BIC**
**Model being tested**						**LL**	**UL**		
**Testing measurement invariance**									
STEP 0: Configural invariance									
White Americans	3,049	344	0.950	0.944	0.036	0.034	0.038	109,824	111,384
Asian Americans	2,666	344	0.926	0.916	0.045	0.042	0.048	52,547	53,843
Chinese	2,416	342	0.932	0.922	0.039	0.036	0.043	50,631	51,922
Japanese	2,721	350	0.937	0.928	0.042	0.039	0.044	61,005	62,402
Taiwanese	2,695	351	0.929	0.919	0.043	0.041	0.046	52,098	53,464
Multigroup fully unconstrained (*N* = 2,091)	13,531	1,732	0.938	0.929	0.040	0.039	0.042	326,091	335,869
STEP 1: Metric invariance	14,311	1,488	0.932	0.924	0.042	0.041	0.043	326,383	334,783
STEP 2: Intercept-only invariance									
FULL: Constraining all item intercepts	16,741	1,488	0.904	0.894	0.049	0.048	0.051	328,813	337,214
PARTIAL: Releasing specific item-group pairs	15,760	1,516	0.915	0.906	0.047	0.046	0.048	327,888	336,446
STEP 3: Scalar invariance (partial)	16,692	1,272	0.907	0.900	0.048	0.047	0.049	328,332	335,513
STEP 4: Full uniqueness invariance	18,720	1,028	0.887	0.882	0.052	0.051	0.053	329,872	335,676
**Examining factor structure (*****N*** **= 1,049)**									
18 subscales loading onto 4 composites	5,282	213	0.922	0.918	0.044	0.043	0.045	168,976	170,031
18 subscales loading onto 1 composite	7,097	207	0.882	0.876	0.054	0.053	0.055	170,779	171,804
61 items loading onto 4 composites	18,490	195	0.631	0.615	0.095	0.094	0.097	182,147	183,114
61 items loading onto a single score	27,067	189	0.442	0.420	0.117	0.116	0.118	190,712	191,649

*# of param = number of parameters estimated in each model*.

### Stability of the TTEA Inventory Factor Structure

Having created the TTEA inventory from a pool of 122 items (across four languages) and then established sufficient measurement invariance to allow scores to be compared across those translations (as the scale predominantly functions consistently across those translations and cultural groups), we then randomly split the entire sample into two halves, conducting hierarchical EFAs in one half (*n* = 1,042; using SPSS 23) and a corresponding CFA in the other half (*n* = 1,049; using Mplus 7.11) to assess the stability of the correlational structure of the TTEA inventory.

#### Exploratory Factor Analyses of the TTEA Inventory

We ran an EFA using principle axis factoring with oblimin rotation (to allow the factors to correlate) on the 61 items of the TTEA. The scree-plot and the Kaiser-Guttman criteria supported extracting the 18 expected factors of the TTEA inventory. This solution accounted for 72% of the variance in the 61 items and yielded strong factor loadings (i.e., structural coefficients ≥0.64) for all of the items on their respective subscales (see Table 4). In contrast, when solutions with fewer factors were attempted, those analyses yielded increasingly large numbers of items with poor (i.e., below 0.4) loadings on their primary factors. We then ran a higher-order EFA (with principle axis factoring and Oblimin rotation) on the TTEA subscale scores. The scree-plot and the Kaiser-Guttman criteria supported extracting four higher-order factors across the subscales of the TTEA (Table 5). As expected, factors representing Buddhism and Taoism emerged. However, instead of supporting one global Confucianism factor, the EFA results suggested two distinct Confucianism factors—one representing three empowering tenets (self-cultivation, leading by example, and human heartedness) and another representing five restrictive tenets/features (propriety pressure, intrinsic propriety, relational hierarchy, interpersonal harmony, and conforming to social norms).

**Table 4:** Correlational structure of the TTEA Inventory using exploratory (EFA) and confirmatory (CFA) factor analyses in separate sample halves.

**Ideology**	**EFA**	**CFA**		**Ideology**	**EFA**	**CFA**	
** *Tenet***		**β**	**SE**	***Tenet***		**β**	**SE**
**Buddhism**				**Taoism**			
***Not Self*** (loading on higher order Buddhism factor)	**0.74**	**0.70**	**0.021**	***Embracing Contradiction*** (loading on higher order Taoism factor)	**0.65**	**0.59**	**0.026**
^*^ Reminded myself that there is no I	0.89	0.88	0.014	^*^ Different points of view can be equally valid	0.88	0.86	0.013
^*^ Recalled that what is me is really a delusion	0.90	0.90	0.014	For important issues, there is usually more than one right answer	0.75	0.66	0.020
***Active Karmic View*** (loading on higher order Buddhism factor)	**0.70**	0.86	0.016	^*^ Even contradicting attitudes or ideas can work together to promote growth	0.79	0.84	0.014
^*^ Strove to behave in a way that would promote positive karma	0.91	0.83	0.014	There are always two sides to everything, depending on how you look at it	0.61	0.60	0.023
^*^ Let my belief in karma push me to be my best possible self	0.89	0.84	0.014	***Wu-Wei/Non-interference*** (loading on higher order Taoism factor)	**0.59**	**0.56**	**0.026**
Recognized that I have control over my behaviors, which leads to karmic repercussions	0.81	0.87	0.012	^*^ Sometimes it's better not to do anything	0.96	0.89	0.011
***Interconnectedness*** (loading on higher order Buddhism factor)	**0.68**	**0.71**	**0.020**	^*^ Often the best course of action might be to do nothing	0.83	0.88	0.011
^*^ I recognized we are all interconnected and go through many of the same situations	0.88	0.83	0.011	Sometimes keeping silent brings about the best result	0.77	0.75	0.016
^*^ I reminded myself that I am not alone but part of a greater whole	0.87	0.88	0.009	***Zi-Ran*** (loading on higher order Taoism factor)	**0.58**	**0.82**	**0.016**
I felt myself as being part of something greater	0.84	0.83	0.011	^*^ I maintain inner peace by accepting things as they come	0.83	0.84	0.012
I considered how I am related to everything	0.87	0.83	0.011	^*^ I try to remain neutral toward others, embracing them where they are	0.77	0.77	0.015
***Meditation*** (loading on higher order Buddhism factor)	**0.60**	**0.68**	**0.021**	I accept myself as I am	0.72	0.73	0.017
Practiced Meditation (i e breathing, walking, chanting, Koans, etc.)	0.92	0.84	0.010	Regardless of how they affect me I accept things as they occur	0.67	0.79	0.014
^*^ Meditated to quiet my mind	0.91	0.86	0.009	***Cyclic Nature*** (loading on higher order Taoism factor)	**0.48**	**0.85**	**0.015**
^*^ Mediated to become more aware	0.93	0.95	0.005	^*^ You can gain harmony in life by understanding and embracing its cycles	0.95	0.86	0.011
Meditated to gain clarity for dealing with my problem	0.92	0.94	0.005	^*^ Life is a cycle, and so peace can only be achieved by enjoying the peaks and valleys without getting overwhelmed by them	0.89	0.85	0.012
***Punishing Karmic View*** (loading on higher order Buddhism factor)	**0.59**	**0.64**	**0.023**	It is possible to find serenity in the midst of change by seeing that change as part of the cycle of life	0.81	0.85	0.011
^*^ I understood I must suffer for my past actions	0.92	0.91	0.009	Opening yourself to the truths inside can bring deeper wisdom and meaning to life	0.76	0.80	0.013
^*^ Realized this is the price I have to pay for my previous actions	0.89	0.87	0.011	***Tranquility*** (loading on higher order Taoism factor)	**0.43**	**0.70**	**0.022**
Believed my bad actions in the past would come back to affect me negatively in this situation	0.81	0.82	0.012	^*^ I remain calm in all situations	0.87	0.87	0.013
				^*^ I like to become still and quiet before I act	0.76	0.71	0.018
				I try to maintain a calm, composed attitude, rather than get emotionally involved in things	0.78	0.82	0.014
**Restrictive Confucianism**				**Empowering Confucianism**			
***Propriety Pressure*** (loading on higher order R-Confucianism)	**0.73**	**0.59**	**0.029**	***Self-cultivation*** (loading on higher order E-Confucianism)	**0.83**	**0.90**	**0.013**
^*^ Feel ashamed when I do not uphold proper social etiquette in public	0.93	0.87	0.018	^*^ I strive to take the higher ground and be the better person	0.87	0.85	0.011
^*^ Feel guilty when I fail to act properly in public	0.88	0.95	0.018	I try to be a virtuous person	0.85	0.84	0.011
***Intrinsic Propriety*** (loading on higher order ideology)	**0.55**	**0.64**	**0.032**	^*^ I try to cultivate the best in myself	0.85	0.85	0.011
^*^ Am conscientious of acting properly in casual social gatherings	0.83	0.74	0.027	I strive to be my best self	0.73	0.75	0.016
^*^ Act properly even when others around me have let loose	0.74	0.78	0.028	***Leading by Example*** (loading on higher order E-Confucianism)	**0.76**	**0.88**	**0.014**
***Relational Hierarchy*** (loading on higher order R-Confucianism)	**0.52**	**0.70**	**0.027**	^*^ I hope that by improving myself I might benefit others around me	0.87	0.85	0.011
^*^ Give more weight to suggestions of people who are older than I am	0.85	0.87	0.011	^*^ By being the best I can be, I hope to inspire others	0.82	0.80	0.014
^*^ Defer to the wisdom of my elders even though I am more educated	0.84	0.84	0.013	I strive to be the type of person that leads by example	0.79	0.81	0.014
Trust the opinion of my elders more than my own	0.79	0.78	0.015	I take personal responsibility in helping to promote the greater social good	0.74	0.79	0.014
Respect the wisdom of elders even if I cannot understand their perspective	0.65	0.52	0.025	***Human Heartedness*** (loading on higher order E-Confucianism)	**0.74**	**0.84**	**0.015**
***Interpersonal Harmony*** (loading on higher order R-Confucianism)	**0.50**	**0.47**	**0.033**	^*^ I strive to be kind and loving to the people in my life	0.87	0.87	0.010
^*^ Keep silent about disagreements to avoid conflict with others	0.82	0.78	0.017	I am always kind to the people in my life	0.80	0.80	0.013
^*^ I would rather suppress my views and opinions if they are in disagreement with others	0.82	0.80	0.016	I am loyal to my family, friends, and work	0.75	0.76	0.015
Avoid discussing topics that might lead to disagreements	0.76	0.74	0.018	^*^ I try to do good for others	0.75	0.81	0.013
Avoid disagreeing with someone to maintain harmony	0.70	0.68	0.020				
***Conforming to Social Norms*** (loading on higher order R-Confucianism)	**0.44**	**0.63**	**0.028**	**CORRELATIONS AMONG IDEOLOGIES**	B	RC	EC
^*^ One should recognize and adhere to social expectations, norms, and practices	0.93	0.89	0.018	Buddhism (B)	–		
One should adhere to the values, beliefs, and behaviors that one's society considers normal and acceptable	0.87	0.80	0.020	Restrictive Confucianism (RC)	0.34	–	
				Empowering Confucianism (EC)	0.36	0.27	–
^*^ Following familial and social expectations is important	0.84	0.89	0.017	Taoism (T)	0.61	0.33	0.49

EFA, Pattern coefficients for each item loading on its respective factor and each subscale loading on its respective ideology from lower-order and higher-order Exploratory Factor Analyses (respectively) conducted in one sample half (n = 1,042). Exploratory Factor Analyses were conducted with principle axis factoring and Oblimin rotation (to allow the extracted factors to correlate). Pattern coefficients for the subscales from the higher order factor analysis have been bolded and placed in parentheses to facilitate interpretation. CFA = Standardized factor loadings from a Confirmatory Factor Analysis conducted in the second sample half (n = 1,049) testing the structure of the 18 tenet subscales organized into 4 higher-order ideologies. This model demonstrated sufficient fit [${\text{χ}}_{\left(1,859\right)}^{2}$ = 5,758, p < 0.001; CFI = 0.918; SRMR = 0.075; RMSEA = 0.045, 90% CI LL = 0.043, UL = 0.046], confirming the correlational structure of the TTEA inventory in the second sample half. The (^*^) indicate the items of the 36-item version of the TTEA inventory.

**Table 5:** Pattern coefficients from higher order EFAs on the TTEA subscale scores conducted in a random sample half (*N* = 1,042).

**Factor** **Subscale**	**Buddhism**	**Empowering Confucianism**	**Restrictive Confucianism**	**Taoism**
**Buddhism**				
Not self	**0.74**	−0.06	−0.02	−0.01
Active karmic view	**0.70**	0.03	0.10	0.13
Interconnectedness	**0.68**	0.30	−0.13	0.06
Meditation	**0.60**	0.06	0.00	0.06
Punishing karmic view	**0.59**	−0.12	0.25	0.10
**Empowering Confucianism**				
Self-cultivation	−0.01	**0.83**	0.06	0.08
Leading by example	0.19	**0.76**	0.02	−0.04
Human heartedness	−0.08	**0.74**	0.08	0.17
**Restrictive Confucianism**				
Propriety pressure	−0.09	−0.02	**0.73**	−0.02
Intrinsic propriety	−0.07	0.31	**0.55**	0.06
Relational hierarchy	0.15	0.13	**0.52**	−0.05
Interpersonal harmony	−0.04	−0.11	**0.50**	0.16
Conforming to social norms	0.21	0.05	**0.44**	−0.07
**Taoism**				
Embracing contradiction	−0.11	0.19	−0.04	**0.65**
Non-interference	0.12	−0.15	0.15	**0.59**
Ziran	0.21	0.20	−0.05	**0.58**
Cyclic nature	0.37	0.09	−0.01	**0.48**
Tranquility	0.27	0.05	0.07	**0.43**
**Correlations among subscales**				
Buddhism	1.00			
Empowering Confucianism	0.28	1.00		
Restrictive Confucianism	0.31	0.18	1.00	
Taoism	0.44	0.37	0.32	1.00

*This higher-order Exploratory Factor Analyses was conducted on TTEA subscales scores within the same sample half (N = 1,042) in which the lower-order factor analysis was conducted. It was conducted using principle axis factoring and Oblimin rotation (to allow the extracted factors to correlate). The strongest loadings of each subscale have been bolded for ease of interpretation*.

#### Confirmatory Factor Analyses of the TTEA Inventory

We then ran a hierarchical CFA in the other sample half to help verify the stability of the TTEA correlational structure. As shown in the bottom half of Table 3, a model mirroring this structure (i.e., 61 items forming 18 tenet subscales, which in turn organize into four ideologies) demonstrated reasonable fit in the second sample half. In contrast, a model testing 18 tenet subscales forming just one higher order (East Asian) ideology factor demonstrated poor fit as did a model testing the 61-items of the TTEA inventory simply forming four ideologies and a model testing the 61-items of the TTEA inventory forming a single dimension of East Asian beliefs. Table 4 presents the CFA path coefficients of the hierarchical CFA model (18-tenets to four-ideologies). The items of the TTEA inventory all displayed strong loadings on their respective latent tenets, and the latent tenet variables displayed strong loadings on their respective ideologies. Thus, the CFA results in the second sample half continued to support the correlational structure of the TTEA inventory.

### Internal Consistency of the TTEA Subscales

We examined Cronbach alpha coefficients for the TTEA subscales and the four composite scores across the five cultural groups, split by gender (Supplementary Table 2). The TTEA subscales had high levels of internal consistency across the 10 resulting demographic groups (average α = 0.85, SD = 0.06, with 95% of the α's falling between 0.70 and 0.95), suggesting that the TTEA scales will generally function well across the five cultural groups and four languages tested, further extending the measurement invariance findings.

### Differences Across Gender, Age, and Cultural Groups

**Figure 1:**
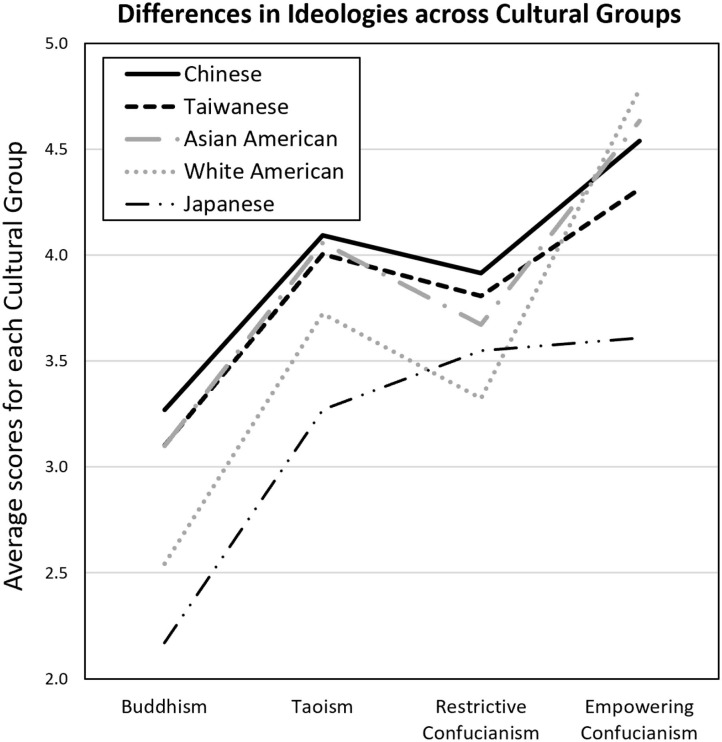
Differences across cultural groups on the four main ideologies assessed with the TTEA Inventory composite scores.

As shown in Table 6, 10 of the 18 TTEA subscales demonstrated significant average differences between men and women in the sample. Most of these differences were fairly modest (Cohen's d's ranging from 0.16 to 0.35), and 8 of the 10 suggested that men endorsed those specific tenets slightly stronger than women. However, the Taoism subscale of embracing contradiction and the empowering Confucianism subscale of human heartedness were endorsed slightly more strongly by women than men. All three empowering Confucianism subscales also demonstrated slightly higher endorsement in individuals younger than 30. Stronger differences emerged on all of the TTEA subscales across the five cultural groups (Cohen's d's between pairs of cultural means showing the greatest differences on each subscale ranged from 0.23 to 1.75). Focusing on the TTEA composites, respondents from Japan were significantly lower on endorsing Buddhist, Taoist, and Empowering Confucianism ideologies, but were comparable to respondents from Taiwan on their fairly high endorsement of Restrictive Confucianism principles (Figure 1). In contrast, White American respondents were significantly lower than most groups on their endorsement of Buddhism, Taoism, and Restrictive Confucianism principles, but had some of the strongest endorsements of the Empowering Confucianism principles (likely as those beliefs are more consistent with trends in the western self-help culture). Asian American respondents also had some of the strongest endorsements of Empowering Confucianism (similar to white Americans), but also strongly endorsed Buddhism and Taoism (similar to Chinese and Taiwanese participants), reflecting more blended ideologies. In contrast to the other groups, the respondents from Taiwan and China were significantly stronger in their endorsements of Restrictive Confucianism tenets.

**Table 6:** Means and standard deviations of the TTEA inventory split by gender, age, and cultural groups.

	**Gender**				**Age**				**Cultural groups**						
	**M**	**W**	**t**	**d**	**<30**	**30+**	**t**	**d**	**Ch**	**Jp**	**Tw**	**AA**	**WA**	**F**	d^**^
**Buddhism**	3.0	2.7	**6.7**	**0.32**	2.8	2.8	1.1	0.05	**3.3**^**A**^	2.2^C^	**3.1**^**A**^	**3.1**^**A**^	2.6^B^	**93.9**	**0.86**
	(1.05)	(1.00)			(1.03)	(1.03)			(0.85)	(0.86)	(0.82)	(1.11)	(1.01)		
Not self	2.5	2.1	**6.6**	**0.31**	2.2	2.3	−0.7	0.03	2.3^B^	1.9^C^	2.4^B^	**2.8**^**A**^	2.1^C^	**29.0**	**0.69**
	(1.34)	(1.28)			(1.34)	(1.28)			(1.27)	(1.11)	(1.21)	(1.47)	(1.31)		
Active karmic view	3.1	2.7	**5.5**	**0.26**	2.8	2.9	−0.3	0.01	**3.7**^**A**^	2.0^D^	**3.5**^**A**^	3.1^B^	2.5^C^	**130.9**	**1.07**
	(1.36)	(1.41)			(1.40)	(1.41)			(1.02)	(1.11)	(0.98)	(1.43)	(1.46)		
Inter-connectedness	3.4	3.2	2.4	0.11	3.3	3.3	0.9	0.04	**3.5**^**AB**^	2.5^C^	**3.5**^**AB**^	**3.6**^**A**^	3.3^B^	**56.1**	**0.92**
	(1.20)	(1.25)			(1.25)	(1.23)			(1.09)	(1.11)	(0.99)	(1.28)	(1.29)		
Meditation	2.9	2.6	**4.7**	**0.22**	2.7	2.6	2.4	0.11	**3.1**^**A**^	2.1^C^	2.6^B^	**3.0**^**A**^	2.6^B^	**29.9**	**0.82**
	(1.44)	(1.33)			(1.35)	(1.39)			(1.31)	(1.13)	(1.36)	(1.53)	(1.34)		
Punishing karmic view	3.1	2.7	**6.5**	**0.31**	2.9	2.8	1.9	0.09	**3.7**^**A**^	2.3^C^	**3.4**^**A**^	3.1^B^	2.4^C^	**103.5**	**1.22**
	(1.27)	(1.37)			(1.36)	(1.34)			(1.09)	(1.20)	(1.02)	(1.38)	(1.34)		
**Taoism**	3.9	3.7	**3.9**	**0.18**	3.8	3.8	1.5	0.06	**4.1**^**A**^	3.3^C^	**4.0**^**A**^	**4.1**^**A**^	3.7^B^	**72.3**	**1.03**
	(0.84)	(0.84)			(0.82)	(0.86)			(0.73)	(0.82)	(0.73)	(0.84)	(0.80)		
Embracing Contradiction	4.2	4.4	**−3.6**	**0.17**	4.4	4.2	**3.9**	**0.17**	**4.5**^**A**^	3.8^B^	**4.4**^**A**^	**4.5**^**A**^	**4.5**^**A**^	**38.7**	**0.71**
	(0.98)	(0.99)			(0.95)	(1.01)			(0.90)	(1.07)	(0.92)	(0.95)	(0.92)		
Non-interference	3.8	3.6	**3.4**	**0.16**	3.7	3.7	0.0	0.00	**4.0**^**A**^	3.3^C^	**4.0**^**A**^	**3.9**^**A**^	3.5^B^	**29.8**	**0.62**
	(1.13)	(1.22)			(1.21)	(1.18)			(1.09)	(1.18)	(1.06)	(1.20)	(1.22)		
Zi-Ran	3.9	3.7	**4.5**	**0.21**	3.8	3.7	1.4	0.06	**4.2**^**A**^	3.1^D^	3.9^B^	**4.1**^**AB**^	3.6^C^	**74.3**	**1.15**
	(1.02)	(1.03)			(1.03)	(1.03)			(0.91)	(1.00)	(0.85)	(1.02)	(0.99)		
Cyclic nature	3.8	3.7	1.0	0.05	3.8	3.7	1.8	0.08	**4.1**^**A**^	3.0^C^	**4.1**^**A**^	**4.0**^**A**^	3.7^B^	**62.4**	**1.01**
	(1.18)	(1.27)			(1.22)	(1.26)			(1.01)	(1.17)	(1.04)	(1.20)	(1.30)		
Tranquility	3.8	3.3	**8.8**	**0.42**	3.5	3.5	−1.2	0.05	**3.7**^**A**^	3.2^B^	**3.6**^**A**^	**3.8**^**A**^	3.3^B^	**24.0**	**0.52**
	(1.07)	(1.14)			(1.17)	(1.10)			(1.08)	(1.08)	(0.99)	(1.21)	(1.16)		
**Restrictive Confucianism**	3.7	3.6	2.4	0.11	3.6	3.6	0.7	0.03	**3.9**^**A**^	3.5^C^	**3.8**^**AB**^	3.7^B^	3.3^D^	**49.5**	**0.64**
	(0.72)	(0.72)			(0.74)	(0.70)			(0.65)	(0.65)	(0.63)	(0.80)	(0.70)		
Propriety pressure	3.7	3.7	0.1	0.00	3.8	3.7	2.5	0.11	**4.2**^**A**^	**4.1**^**A**^	**4.1**^**A**^	3.5^B^	3.2^C^	**62.6**	**0.86**
	(1.23)	(1.27)			(1.29)	(1.23)			(1.21)	(1.09)	(1.07)	(1.31)	(1.24)		
Intrinsic propriety	3.8	3.9	−1.4	0.07	3.9	3.8	1.1	0.05	**4.0**^**A**^	3.4^B^	**3.9**^**A**^	**4.0**^**A**^	**3.9**^**A**^	**25.9**	**0.59**
	(1.00)	(1.06)			(1.06)	(1.03)			(0.99)	(1.03)	(0.93)	(1.11)	(1.03)		
Relational hierarchy	3.3	3.2	2.3	0.11	3.3	3.2	1.5	0.07	**3.5**^**A**^	3.1^B^	3.2^B^	**3.6**^**A**^	3.1^B^	**25.5**	**0.50**
	(0.99)	(0.92)			(0.95)	(0.93)			(0.88)	(0.91)	(0.84)	(1.06)	(0.92)		
Interpersonal harmony	3.4	3.4	0.0	0.00	3.4	3.4	0.0	0.00	**3.3**^**AB**^	**3.4**^**AB**^	**3.5**^**A**^	**3.5**^**A**^	3.3^B^	**5.8**	**0.23**
	(0.90)	(0.92)			(0.93)	(0.88)			(0.80)	(0.87)	(0.84)	(1.05)	(0.93)		
Conforming to social norms	4.1	3.7	**7.0**	**0.33**	3.7	3.9	**−3.0**	**0.13**	**4.5**^**A**^	3.7^C^	**4.4**^**A**^	3.9^B^	3.2^D^	**117.6**	**1.08**
	(1.04)	(1.14)			(1.16)	(1.09)			(0.93)	(0.95)	(0.90)	(1.13)	(1.10)		
**Empowering Confucianism**	4.3	4.5	**−3.4**	**0.16**	4.5	4.3	**4.5**	**0.20**	4.5^B^	3.6^D^	4.3^C^	**4.6**^**AB**^	**4.8**^**A**^	**149.3**	**1.47**
	(0.91)	(0.89)			(0.87)	(0.91)			(0.78)	(0.87)	(0.73)	(0.84)	(0.76)		
Self-cultivation	4.4	4.5	−2.7	0.13	4.6	4.4	**4.5**	**0.20**	4.6^B^	3.5^D^	4.4^C^	**4.7**^**AB**^	**4.9**^**A**^	**157.3**	**1.56**
	(1.03)	(0.99)			(0.97)	(1.04)			(0.91)	(0.98)	(0.85)	(0.90)	(0.81)		
Leading by example	4.1	4.2	−2.3	0.11	4.3	4.0	**5.0**	**0.22**	4.2^EC^	3.3^D^	4.0^C^	**4.4**^**AB**^	**4.5**^**A**^	**91.3**	**1.13**
	(1.09)	(1.13)			(1.10)	(1.12)			(0.99)	(1.06)	(0.92)	(1.08)	(1.07)		
Human heartedness	4.5	4.7	**−4.4**	**0.21**	4.5	4.7	**2.3**	**0.20**	4.7^B^	4.0^D^	4.5^C^	4.8^B^	**5.0**^**A**^	**97.0**	**1.17**
	(0.94)	(0.89)			(0.94)	(0.89)			(0.84)	(0.94)	(0.78)	(0.89)	(0.76)		

*Means are presented on the first row of each scale and standard deviations are presented in parentheses on the second row. t = t-tests for independent groups testing for differences by gender and age groups. d = Cohen's d estimates for pairs of means*.

** *d = Cohen's d estimates for the pairs of cultural means showing the biggest difference. t-tests significant at p < 0.05 (and their corresponding Cohen's d values) have been bolded for ease of interpretation. F = a main effect for Cultural group from an ANOVA run on each scale. F's significant at p < 0.05 have been bolded. Any significant main effects were followed up with Tukey post-hoc analyses to identify significant differences across specific culture groups. Those post-hoc results are presented in the superscripted letters following each mean with significantly different means having different superscripted letters. The highest culture group means have been bolded in each row to facilitate interpretation*.

### Discriminant Validity Within the TTEA Inventory

Table 7 presents the correlations among the tenet subscales and the higher-order composite scales of the TTEA inventory. As can be seen in the table, the subscales corresponding to each higher-order ideology demonstrated moderate correlations with one another, suggesting that although they share sufficient variance to be collapsed into more general composites (i.e., Buddhism, Taoism, Restrictive Confucianism, and Empowering Confucianism), each tenet subscale also offers researchers unique variance for understanding different cultural points of view. Although the correlations among tenets were the strongest within tenets assessing the same higher-order ideology, the correlations in Table 7 also suggest that some tenets, for example cyclic nature, correlated modestly with other ideologies (i.e., Buddhism and Empowering Confucianism). This is possibly due to the cross-pollination of these ideologies within East Asia.

**Table 7:** Correlations among the TTEA subscales.

	**Ideology composite**	**Correlations among TTEA subscales**																						
		**Tenet subscales**	**B**	**B1**	**B2**	**B3**	**B4**	**B5**	**T**	**T1**	**T2**	**T3**	**T4**	**T5**	**RC**	**RC1**	**RC2**	**RC3**	**RC4**	**RC5**	**EC**	**EC1**	**EC2**	**EC3**
**B**	**Buddhism**			**0.78**	**0.85**	**0.78**	**0.81**	**0.75**	**0.59**	0.29	0.42	0.48	**0.62**	0.47	0.38	0.14	0.27	0.38	0.26	0.30	0.42	0.35	0.45	0.31
	B1	Not self	**0.75**		**0.52**	**0.57**	**0.54**	0.45	0.34	0.07	0.23	0.29	0.37	0.34	0.18	−0.02	0.12	0.25	0.20	0.13	0.20	0.14	0.27	0.12
	B2	Active karmic view	**0.83**	0.47		**0.59**	**0.62**	**0.65**	**0.58**	0.32	0.44	0.45	**0.60**	0.41	0.37	0.17	0.25	0.34	0.23	0.33	0.40	0.35	0.41	0.31
	B3	Interconnectedness	**0.74**	**0.55**	**0.51**		**0.55**	0.44	**0.53**	0.31	0.32	0.49	**0.53**	0.40	0.28	0.06	0.26	0.30	0.13	0.26	**0.51**	0.43	**0.52**	0.42
	B4	Meditation	**0.70**	0.40	0.48	0.44		0.45	**0.50**	0.21	0.38	0.39	**0.53**	0.43	0.27	0.10	0.22	0.30	0.17	0.17	0.32	0.27	0.35	0.23
	B5	Punishing karmic view	**0.73**	0.43	**0.62**	0.33	0.33		0.40	0.24	0.31	0.30	0.43	0.28	0.41	0.27	0.24	0.34	0.28	0.32	0.25	0.21	0.26	0.19
**T**	**Taoism**		**0.61**	0.40	**0.55**	**0.52**	0.42	0.40		**0.74**	**0.74**	**0.83**	**0.82**	**0.76**	0.40	0.19	0.34	0.32	0.25	0.30	**0.59**	**0.53**	**0.51**	**0.53**
	T1	Embracing contradiction	0.29	0.14	0.28	0.26	0.20	0.20	**0.66**		0.42	**0.58**	**0.54**	0.39	0.30	0.21	0.30	0.19	0.15	0.19	0.49	0.44	0.40	0.48
	T2	Non-interference	0.39	0.27	0.34	0.27	0.25	0.33	**0.71**	0.34		**0.51**	0.47	0.48	0.31	0.17	0.26	0.22	0.25	0.21	0.31	0.28	0.27	0.29
	T3	Zi-Ran	0.49	0.32	0.46	0.48	0.31	0.26	**0.79**	0.44	0.42		**0.61**	**0.57**	0.34	0.15	0.31	0.30	0.17	0.28	**0.57**	**0.54**	0.48	**0.52**
	T4	Cyclic nature	**0.61**	0.39	**0.56**	**0.52**	0.43	0.39	**0.80**	0.49	0.40	**0.55**		**0.52**	0.32	0.14	0.25	0.28	0.19	0.29	0.49	0.43	0.46	0.41
	T5	Tranquility	0.45	0.34	0.37	0.40	0.32	0.27	**0.73**	0.27	0.41	**0.56**	0.46		0.27	0.08	0.24	0.27	0.19	0.19	0.43	0.39	0.40	0.38
**RC**	**Restrictive Confucianism**		0.32	0.20	0.31	0.13	0.18	0.36	0.29	0.11	0.29	0.18	0.19	0.26		**0.77**	**0.71**	**0.71**	**0.61**	**0.67**	0.39	0.36	0.34	0.35
	RC1	Propriety pressure	0.13	0.04	0.15	−0.03	0.06	0.23	0.10	0.05	0.14	−0.01	0.06	0.09	**0.75**		0.49	0.34	0.35	0.38	0.18	0.16	0.14	0.18
	RC2	Intrinsic propriety	0.22	0.12	0.24	0.14	0.14	0.19	0.24	0.16	0.19	0.15	0.20	0.19	**0.68**	0.44		0.39	0.31	0.26	0.42	0.44	0.34	0.36
	RC3	Relational hierarchy	0.33	0.26	0.27	0.22	0.20	0.28	0.29	0.11	0.19	0.26	0.20	0.29	**0.66**	0.28	0.32		0.34	0.45	0.33	0.28	0.32	0.30
	RC4	Interpersonal harmony	0.13	0.11	0.08	0.00	0.07	0.21	0.17	0.07	0.25	0.05	0.09	0.16	**0.58**	0.31	0.27	0.32		0.23	0.09	0.09	0.09	0.07
	RC5	Conforming to social norms	0.28	0.19	0.31	0.12	0.14	0.28	0.20	0.00	0.21	0.19	0.13	0.19	**0.66**	0.36	0.25	0.38	0.18		0.34	0.29	0.30	0.33
**EC**	**Empowering Confucianism**		0.36	0.15	0.33	0.47	0.30	0.11	0.46	0.38	0.18	0.44	0.42	0.29	0.22	0.04	0.37	0.21	0.02	0.11		**0.90**	**0.90**	**0.88**
	EC1	Self-cultivation	0.31	0.11	0.28	0.39	0.28	0.10	0.42	0.36	0.17	0.38	0.38	0.27	0.21	0.05	0.38	0.18	0.03	0.07	**0.90**		**0.70**	**0.71**
	EC2	Leading by example	0.39	0.20	0.34	**0.50**	0.31	0.14	0.41	0.30	0.15	0.39	0.41	0.28	0.17	0.03	0.28	0.19	−0.01	0.10	**0.90**	**0.71**		**0.69**
	EC3	Human heartedness	0.23	0.08	0.22	0.34	0.20	0.04	0.40	0.36	0.17	0.40	0.34	0.23	0.21	0.03	0.33	0.21	0.05	0.11	**0.86**	**0.69**	**0.63**	

*Correlations in men and women are presented above and below the diagonal, respectively. Correlations above 0.10 in men and above 0.07 in women were significant at p < 0.01. For ease of interpretation, correlations above 0.5 have been bolded*.

### Discriminant Validity of the TTEA Inventory From Counseling Attitudes, Well-Being, and Mindfulness

As shown in Table 8, the TTEA subscales demonstrated only low to modest correlations with a large set of conceptually related yet distinct constructs from the nomological net from the cross-cultural literature. Specifically, the ideologies of Buddhism, Taoism, and Restrictive Confucianism (along with a majority of their individual tenets) were weakly associated with feeling less embarrassment at the prospect of seeking professional mental health treatment, suggesting that adopting traditional East Asian tenets (e.g., not-self, active karmic view, tranquility) into one's daily life might help to slightly reduce the internalization of cultural stigmas against counseling. However, those three ideologies were largely uncorrelated with positive counseling attitudes. In contrast, higher endorsement of the tenets of empowering Confucianism was associated with stronger positive attitudes toward counseling, suggesting potential points of intervention for promoting treatment seeking. Turning to the remaining correlations, Buddhism, Taoism, and Empowering Confucianism were all correlated with greater mindfulness and life satisfaction. In contrast, Restrictive Confucianism was correlated with greater psychological distress, whereas Empowering Confucianism was correlated with lower psychological distress. Finally, both Buddhism and Empowering Confucianism were correlated with higher somatic anxiety. All of the dimensions of the TTEA inventory showed significant correlations with culturally informed behaviors like collectivism, filial piety, and face concerns, highlighting how such tenets might inform behavior and individual functioning across various cultures. Taken together, the generally low to modest size of the correlations in Table 8 suggest that the TTEA inventory is assessing a set of tenets that is distinct from related constructs like collectivism and mindfulness, underscoring its potential unique contribution to the literature.

**Table 8:** Correlations with existing scales to demonstrate discriminant validity.

**Existing scale**	**Discriminant validity correlations between ttea subscales and distinct constructs**																						
	**Subscale**	**B**	**B1**	**B2**	**B3**	**B4**	**B5**	**T**	**T1**	**T2**	**T3**	**T4**	**T5**	**RC**	**RC1**	**RC2**	**RC3**	**RC4**	**RC5**	**EC**	**EC1**	**EC2**	**EC3**
		**Buddhism composite**	**Not self**	**Active karmic view**	**Interconnectedness**	**Meditation**	**Punishing karmic view**	**Taoism composite**	**Embracing contradiction**	**Non-interference**	**Ziran**	**Cyclic nature**	**Tranquility**	**Restrictive Confucianism**	**Propriety pressure**	**Intrinsic propriety**	**Relational hierarchy**	**Interpersonal harmony**	**Conforming to norms**	**Empowering Confucianism**	**Self-cultivation**	**Leading by example**	**Human heartedness**
**Individual functioning**																							
	Positive counseling attitude	0.07	−0.03	0.04	0.15	0.14	−0.02	0.19	**0.31**	0.07	0.14	0.20	0.02	0.02	−0.01	0.17	0.03	0.01	−0.10	**0.37**	**0.34**	**0.30**	**0.34**
	Counseling embarrassment	−0.27	−0.25	−0.22	−0.13	−0.15	−0.27	−0.18	−0.02	−0.16	−0.14	−0.13	−0.20	−0.26	−0.11	−0.16	−0.24	−0.20	−0.20	−0.04	−0.06	−0.03	−0.01
	Mindfulness (PMA)	**0.53**	**0.31**	**0.42**	**0.51**	**0.50**	0.30	**0.53**	**0.40**	0.30	**0.43**	**0.51**	**0.36**	0.14	0.04	0.20	0.14	0.07	0.03	**0.48**	**0.43**	**0.44**	**0.39**
	Inattentive/unaware	0.20	0.20	0.17	0.03	0.12	0.26	0.14	0.04	0.19	0.05	0.08	0.13	0.19	0.16	0.04	0.15	0.19	0.13	−0.05	−0.07	−0.02	−0.05
	Judging thoughts/feelings	0.25	0.16	0.23	0.05	0.15	**0.37**	0.14	0.11	0.19	0.00	0.13	0.06	**0.34**	**0.32**	0.17	0.21	0.26	0.19	0.05	0.05	0.06	0.03
	Non-reactivity	**0.44**	**0.31**	**0.38**	**0.44**	**0.36**	0.20	**0.53**	0.23	**0.31**	**0.54**	**0.39**	**0.51**	0.13	−0.01	0.11	0.17	0.00	0.18	**0.32**	0.29	0.29	0.27
	Peace of mind	**0.35**	0.26	0.29	**0.40**	0.30	0.10	**0.44**	0.20	0.25	**0.49**	**0.33**	**0.39**	0.08	−0.05	0.07	0.17	−0.04	0.16	**0.35**	**0.33**	**0.30**	**0.31**
	Life SATISFACTION	0.29	0.23	0.22	**0.37**	0.26	0.03	**0.36**	0.19	0.18	**0.40**	0.28	**0.30**	0.05	−0.10	0.07	0.16	−0.04	0.09	**0.37**	**0.34**	**0.34**	**0.32**
	Psychological distress	0.05	0.04	0.03	−0.14	0.03	0.24	−0.03	0.03	0.08	−0.17	0.01	−0.08	0.15	0.21	0.09	0.05	0.21	−0.04	−0.11	−0.10	−0.09	−0.12
	Somatic anxiety	0.20	0.19	0.12	0.04	0.17	0.23	0.06	−0.01	0.11	−0.05	0.06	0.07	0.13	0.14	0.08	0.11	0.14	−0.02	−0.07	−0.07	−0.03	−0.11
Self-identified religious/spiritual affiliation^*^																							
	Christian	0.03	0.06	−0.03	0.11	0.01	−0.04	0.02	0.02	0.01	0.05	−0.01	0.02	0.01	−0.13	0.11	0.09	−0.01	0.00	0.20	0.18	0.19	0.17
	Buddhist	0.12	0.11	0.10	0.03	0.12	0.10	0.00	−0.08	0.03	−0.03	0.02	0.04	0.11	0.13	−0.04	0.10	0.04	0.14	−0.14	−0.16	−0.12	−0.11
	Taoist	0.13	0.07	0.16	0.07	0.06	0.14	0.12	0.06	0.08	0.10	0.10	0.09	0.12	0.09	0.05	0.04	0.02	0.18	0.00	0.01	−0.01	−0.01
	Atheist	−0.16	−0.14	−0.10	−0.17	−0.13	−0.06	−0.08	−0.03	−0.06	−0.04	−0.10	−0.05	−0.07	0.03	−0.07	−0.12	−0.04	−0.07	−0.11	−0.08	−0.11	−0.11
**Culturally informed behaviors**																							
	Collectivism	**0.48**	**0.34**	**0.44**	**0.34**	**0.33**	**0.39**	**0.44**	0.26	0.28	**0.38**	**0.39**	**0.33**	**0.42**	0.22	0.25	**0.37**	0.22	**0.37**	**0.36**	**0.31**	**0.33**	**0.31**
	Reciprocal filial piety	**0.46**	**0.32**	**0.44**	**0.32**	0.28	**0.38**	**0.36**	0.14	0.26	**0.34**	**0.30**	**0.30**	**0.37**	0.21	0.16	**0.35**	0.13	**0.41**	0.20	0.15	0.20	0.17
	Authoritarian filial piety	**0.55**	**0.46**	**0.49**	**0.34**	**0.39**	**0.45**	**0.36**	0.06	0.29	**0.33**	**0.30**	**0.34**	**0.42**	0.21	0.18	**0.43**	0.21	**0.40**	0.14	0.12	0.17	0.08
	Face concerns for self	**0.36**	0.17	**0.39**	0.23	0.20	**0.37**	**0.35**	0.23	0.25	0.23	**0.33**	0.25	**0.43**	**0.33**	0.30	0.25	0.23	**0.34**	**0.32**	**0.31**	0.29	0.24
	Face concerns for other	**0.40**	0.20	**0.39**	0.25	0.26	**0.41**	**0.43**	0.28	**0.33**	**0.30**	**0.35**	**0.33**	**0.52**	**0.37**	**0.36**	**0.33**	**0.35**	**0.34**	**0.30**	0.29	0.26	0.25

*Correlations presented in this table were calculated in the entire sample. All correlations with absolute magnitudes ≥ 0.073 were statistically significant at p < 0.001. For ease of interpretation, correlations with absolute magnitudes ≥ 0.3 have been bolded to facilitate interpretation*.

* *These variables were dichotomous, coding the religious affiliation of each subject*.

### Incremental Predictive Utility of the TTEA Inventory

To evaluate how the TTEA inventory might provide insights into models of individual well-being, we ran regressions predicting current vitality, life satisfaction, and collectivism using the TTEA composite scores as simultaneous predictors (Model 1's) and models using the 18 subscales as simultaneous predictors (Model 2's) of each outcome. The Model 1 results suggested acceptable levels of collinearity among the four composites of the TTEA inventory as the Variance Inflation Factors for those predictors ranged from 1.18 to 1.86 (well below the thresholds of 5.0 or 10.0 to identify problematic levels of collinearity; e.g., Menard, 1995; see O'brien, 2007 for a discussion of accepted thresholds). As shown in the first block of rows of Table 9, each of the four ideologies assessed by the TTEA inventory was uniquely predictive of current levels of vitality (i.e., positive mood and levels of energy), levels of overall life satisfaction, and collectivism. Thus, higher endorsement of Buddhist, Taoist, and empowering Confucian beliefs were each uniquely linked to higher vitality and life satisfaction highlighting the potential life-enriching benefits of those ideologies. In contrast, greater endorsement of restrictive Confucian beliefs was uniquely linked to lower vitality and life satisfaction, suggesting the potential costs of subjugating one's own needs for those of society as a greater whole. Although greater endorsement of each of the four ideologies uniquely predicted greater levels of collectivism, Buddhism and Restrictive Confucianism emerged as particularly strong predictors.

**Table 9:** Multiple regressions predicting individual well-being and collectivism.

**Model**	**Regression results**											
***Ideologies***	**Predicting vitality**				**Predicting life satisfaction**				**Predicting collectivism**			
**Tenets**	**B**	**SE**	**β**	***p***	**B**	**SE**	**β**	***p***	**B**	**SE**	**β**	***p***
**Model 1—Ideologies as predictors**												
(constant)	0.37	0.157		0.019	0.69	0.166		<0.0005	−0.99	0.152		<0.0005
Buddhism	**0.20**	0.030	**0.16**	<0.0005	**0.14**	0.032	**0.11**	<0.0005	**0.33**	0.029	**0.26**	<0.0005
Taoism	**0.34**	0.039	**0.22**	<0.0005	**0.31**	0.041	**0.20**	<0.0005	**0.22**	0.038	**0.14**	<0.0005
Restrictive Confucianism	**−0.28**	0.036	**−0.15**	<0.0005	**−0.23**	0.038	**−0.13**	<0.0005	**0.45**	0.035	**0.25**	<0.0005
Empowering Confucianism	**0.46**	0.031	**0.32**	<0.0005	**0.39**	0.033	**0.27**	<0.0005	**0.18**	0.030	**0.13**	<0.0005
**Model 2—Tenets as predictors**												
(constant)	0.56	0.162		<0.0005	0.72	0.174		<0.0005	−1.07	0.165		<0.0005
***Buddhism***												
Not self	0.00	0.023	0.00	0.882	**0.08**	0.025	**0.08**	0.002	**0.11**	0.024	**0.11**	<0.0005
Active Karma	0.05	0.026	0.06	0.047	−0.01	0.028	0.00	0.787	0.06	0.026	0.07	0.016
Interconnectedness	**0.17**	0.027	**0.16**	<0.0005	**0.14**	0.029	**0.14**	<0.0005	−0.05	0.028	−0.05	0.078
Meditation	**0.09**	0.021	**0.09**	<0.0005	**0.08**	0.023	**0.08**	0.001	0.04	0.021	0.04	0.061
Punishing Karma	**−0.12**	0.023	**−0.12**	<0.0005	**−0.16**	0.025	**−0.17**	<0.0005	**0.11**	0.024	**0.11**	<0.0005
***Taoism***												
Embracing contradiction	−0.04	0.029	−0.03	0.184	−0.04	0.031	−0.03	0.162	0.04	0.030	0.03	0.200
Non-interference	0.03	0.023	0.03	0.222	0.03	0.025	0.02	0.298	0.00	0.024	0.00	0.837
Ziran	**0.30**	0.033	**0.24**	<0.0005	**0.25**	0.035	**0.20**	<0.0005	**0.10**	0.033	**0.08**	0.004
Cyclic nature	−0.02	0.027	−0.02	0.462	−0.01	0.029	−0.01	0.757	**0.08**	0.027	**0.08**	0.004
Tranquility	0.06	0.026	0.05	0.026	**0.08**	0.028	**0.07**	0.004	0.03	0.026	0.02	0.341
***Restrictive Confucianism***												
Propriety pressure	**−0.11**	0.022	**−0.11**	<0.0005	**−0.09**	0.024	**−0.09**	<0.0005	0.05	0.022	0.05	0.037
Intrinsic propriety	−0.04	0.027	−0.04	0.111	−0.07	0.029	−0.06	0.018	−0.02	0.028	−0.02	0.504
Relational hierarchy	0.06	0.029	0.05	0.029	**0.09**	0.031	**0.07**	0.004	**0.16**	0.030	**0.11**	<0.0005
Interpersonal harmony	**−0.15**	0.028	**−0.11**	<0.0005	−0.07	0.030	−0.05	0.017	**0.08**	0.029	**0.06**	0.004
Conforming to social norms	**0.07**	0.024	**0.06**	0.003	0.03	0.026	0.03	0.192	**0.18**	0.024	**0.16**	<0.0005
***Empowering Confucianism***												
Self-cultivation	**0.21**	0.037	**0.17**	<0.0005	**0.16**	0.040	**0.13**	<0.0005	0.06	0.038	0.04	0.144
Leading by example	0.04	0.032	0.03	0.218	0.03	0.034	0.03	0.350	0.08	0.033	0.07	0.018
Human heartedness	0.08	0.038	0.06	0.034	**0.11**	0.041	**0.08**	0.007	**0.12**	0.039	**0.08**	0.003

*Regression coefficients significant at ≤ 0.01 have been bolded for ease of interpretation*.

As shown in the lower portion of Table 9, when the 18 TTEA subscales were used as predictors for these outcomes, a far more nuanced pattern of findings emerged. Although collinearity among the 18 TTEA subscales was slightly higher (VIFs ranging from 1.28 to 2.78), they remained within an acceptable range suggesting that the individual tenet subscales each had meaningful unique variance to contribute to the prediction of individual well-being and collectivism. Within these models, Ziran (i.e., accepting things as they are), interconnectedness, and self-cultivation emerged as some of the stronger positive predictors of both measures of individual well-being, seemingly promoting both greater vitality and a strong sense of satisfaction with life, whereas punishing karma and propriety pressure were predictive of lower vitality and life satisfaction. However, unique predictive associations also emerged for both individual well-being outcomes. Thus, when predicting vitality, interpersonal harmony was uniquely predictive of lower vitality whereas conforming to social norms was predictive of slightly higher vitality although neither of those tenets emerged as significant predictors of life satisfaction. In contrast, when predicting life satisfaction, not self (i.e., not clinging to one's sense of ‘self') and relational hierarchy both predicted slightly higher life satisfaction although neither were uniquely predictive of current vitality. Turning to the prediction of collectivism, conforming to social norms emerged as a particularly strong predictor, as did not self, punishing karma, and relational hierarchy, thereby highlighting the aspects of collectivism that require individuals to place the needs of others before their own. These findings begin to highlight the potential utility of the TTEA inventory to shed light on various cultural differences across a wide range of behaviors and outcomes, as they demonstrate how the measure offers researchers 18 individual tenets and four larger composites as possible mechanisms to begin to characterize the rich ideological fabric underlying various cultures.

## Discussion

The TTEA inventory represents a conceptually grounded and comprehensive measure assessing 18 different tenets of East Asian thought—not only integrating work from previous scales but also offering a concrete method of representing the larger ideologies of Buddhism, Taoism, and Confucianism within cultural models. The TTEA inventory therefore provides a broad conceptual framework for future research on East Asian culture.

### Implications

#### TTEA Represents a Truly Novel Assessment Tool

Most previous measurement work in this area focused on creating single-dimension broad-band scales to assess a general East Asian worldview (e.g., the Asian Values Scale; Kim et al., 1999) or on the main societal results of those Eastern ideologies (e.g., collectivism: Hui, 1988). More recently, researchers have developed more conceptually-focused scales assessing single tenets (e.g., naïve dialecticism) or small sets of tenets representing an ideology (e.g., Buddhist Coping Measure; BCOPE; Phillips et al., 2012). The TTEA inventory instead offers a unifying conceptual framework not only grounded in the foremost East Asian philosophies, but also assessing unique tenets specific to each of them. The TTEA inventory therefore not only clarifies the ideological structure of Buddhism, Taoism, Restrictive, and Empowering Confucianism, but synthesizes these tenets into a cohesive framework illustrating the dynamics among these metaphysical ideas.

#### TTEA Inventory Distinguishes the Restrictive and Empowering Aspects of Confucianism

With over a thousand citations, the single-dimension AVS has dominated research on East Asian values, focusing that research primarily on tenets related to Confucianism assessed by the AVS (Kim et al., 1999; Kim and Hong, 2004). Research on the AVS has yielded mixed results, sometimes uncovering links to higher functioning (Kim and Omizo, 2005), to lower functioning (e.g., Hovey et al., 2006; Iwamoto and Liu, 2010), or failing to find significant associations with individual functioning (e.g., Iwamoto et al., 2010; Miller et al., 2011). The TTEA inventory offers a method of potentially clarifying those mixed results by deconstructing Confucianism into two broader domains (restrictive vs. empowering Confucianism) and further down into eight specific tenets. In fact, the results with the TTEA inventory suggest that the two Confucian composites and the eight Confucian tenets can show meaningfully different patterns of correlation. Thus, the mixed results with the AVS are likely being driven by particularly strong associations between specific outcomes and specific components of Confucianism. At a broader level, the TTEA inventory distinguishing between the self-denying, restrictive aspects of Confucianism (i.e., Restrictive Confucianism) and the self-fulfilling, aspirational aspects of Confucianism (i.e., Empowering Confucianism) offers a key conceptual distinction to advance research in this area. This distinction aligns with the arguments of Hwang (see Hwang, 2001 for a review) who suggests that the ethics of Confucianism could be practiced in two different ways. One form of practicing Confucianism is targeted toward ordinary people as it encourages them to restrict their self-centered impulses (i.e., restrictive Confucianism). The other form discussed by Hwang is targeted toward Jun-Zi (君子) or ideal (i.e., “upright and noble”) people encompassing the moral and ethical qualities described by Confucius. Thus, the second form of practicing Confucianism focuses on encouraging upright and noble persons to pursue self-fulfillment and self-actualization (i.e., empowering Confucianism). The current findings suggest that these two distinct aspects of Confucianism are not opposite to each other like the two ends of one continuum. Instead, these two ideologies should be viewed as distinct yet related, coexisting alongside one another in individuals' moral approaches to life. As the TTEA inventory is the first scale to operationalize these two distinct aspects of Confucianism within a self-report scale, the inventory allows researchers to quantify and distinguish the unique natures of those two domains within models of functioning. In fact, the Empowering Confucianism subscales represent entirely new additions to the literature, offering researchers the first cross-culturally validated measures of self-cultivation, leading by example, and human heartedness.

#### The TTEA Inventory Can Be a Fine-Grained Tool

Given the hierarchical structure of the TTEA inventory, researchers and clinicians have a range of options when using the scale in their work. A researcher or clinician interested in understanding more fine-grained or detail-oriented links between ideological tenets and specific behaviors or outcomes would likely examine the 18 individual subscales separately. In the case of clinicians, this could take the form of an ideological profile for an individual client to help guide therapy with that client. Given the documented disparities in mental health treatment seeking and treatment dropout (e.g., Leong et al., 2001) within Asian populations, use of the TTEA in clinical research could help to clarify some of the more specific ideological barriers to therapy in Asian populations. For researchers, use of the TTEA inventory could take the form of treating those 18 subscale scores as separate constructs in models of individual functioning. For example, as both Buddhism and Taoism offer specific lenses for viewing life, daily stress, and one's place within nature and the broader universe (e.g., Yip, 2005; Chen, 2006), the specific tenets within those larger ideologies will likely show robust links to adaptive processes like mindfulness, emotion regulation, psychological flexibility, stress management and coping, and resilience. The TTEA inventory would provide both an optimized measure as well as a conceptual framework for such investigations. Consistent with this, the individual tenets demonstrated distinct patterns of correlation with dimensions of counseling attitudes, highlighting the rich tapestry of results that can emerge from use of the TTEA subscales.

#### The TTEA Inventory Can Offer a Broader View

After examining the specific tenets assessed by the inventory, clinicians and researchers could also create larger composite scores representing overall levels of Buddhism, Taoism, Restrictive, and Empowering Confucianism within specific clients or within their models. This extends work on constructs like collectivism (e.g., Triandis, 1995), filial piety (e.g., Yeh and Bedford, 2003), and face management (e.g., Oetzel and Ting-Toomey, 2003) by linking those culturally-informed behaviors to the larger East Asian ideologies that underlie those social conventions. For example, whereas Empowering Confucianism offers insights toward becoming a virtuous and self-actualized person, Restrictive Confucianism seeks to promote social order by encouraging individuals to deny personal needs in certain ways to benefit the community. Thus, our results suggest that the four composites would likely demonstrate distinct patterns of association with culturally informed behaviors. The TTEA inventory therefore offers a method of distilling the widely-varying cultural backgrounds of all subjects (both East-Asian as well as individuals from Western cultures who might have sought out Eastern ideologies and teachings to varying degrees) into useful sketches formed by four fundamental scores on the overarching ideologies.

#### The TTEA Inventory Offers a Flexible Tool

For researchers interested in deconstructing the more specific tenets driving specific behaviors or underlying cultural differences, the 61-item TTEA inventory offers researchers and clinicians a comprehensive tool for evaluating and deconstructing cultural differences, by providing four composite scores and 18 individual tenet scores to be examined as possible mechanisms. However, not all future studies might have need of that level of precision. Studies primarily interested in assessing the four main composite ideologies identified in the current work could therefore make use of the 36-item version of the scale. Extending this discussion of options, future researchers and studies primarily interested in assessing Buddhist ideology as a possible mechanism, for example, could simply include the full 16 items assessing the five Buddhism tenets or even just the shorter 10-item version of those five subscales from the 36-item TTEA inventory. Similarly, researchers interested in empowering Confucianism (a set of tenets likely to be linked to the self-help movement) could simply include the 12 items of those corresponding tenet subscales from the full TTEA or even just the six items from those subscales of the 36-item TTEA inventory. Thus, the TTEA inventory could be considered a modular tool, allowing future researchers to use as many or as few of its composites and subscales as are relevant to their projects.

#### Ideologies Showed Meaningful Differences Across Cultural Groups

As shown in Table 6 and Figure 1, TTEA inventory demonstrated clear cross-cultural differences among the East Asian and American cultural groups examined. For instance, despite slight differences, Chinese and Taiwanese respondents demonstrated a very similar trend of endorsing these ideologies. That might illustrate that after decades of separation due to political and historical reasons, these two populations might still share a very similar mentality rooted in traditional Chinese culture. Interestingly, Asian Americans also looked similar to Chinese and Taiwanese respondents on Buddhism and Taoism, highlighting the potential power of enculturation (i.e., adopting the beliefs of your parents and family). Shifting the focus to people living in America, the higher scores on Empowering Confucianism among White and Asian Americans suggest that this specific ideology aligns well with a Western Worldview and highlights that East-Asian framework could still be relevant for understanding Western mentalities. Thus, the TTEA inventory could serve as a tool to help the field of psychology moving from an overly Westernized and WEIRD point of view (Henrich et al., 2010) to a more culturally and ideologically inclusive framework. Finally, Japanese respondents demonstrated a notably different pattern with lower average scores in comparison to the rest of the cultural groups. These findings are consistent with previous findings suggesting that Japanese tend to provide modest self-evaluation and correspondingly lower scores in self-reported studies (Iwata and Buka, 2002; Nishikawa et al., 2016). However, response biases have also been found within Chinese samples (e.g., Chen et al., 1995) and in international samples spanning over 40 distinct countries (Schimmack et al., 2005), and yet the lower means emerged only within the Japanese respondents in the current study. In addition, the measurement invariance analyses suggested that the TTEA subscales were reasonably invariance across the five cultural groups. Thus, the lower average scores observed among the Japanese respondents might represent a unique mentality rather than a simple response bias. Their relatively higher scores on both Confucianism scales also align with the strong emphasis on social order and following norms in Japanese society (Cousins, 1989), and suggest a more secular focus to their mentalities.

#### Ideological Differences Might Explain Other Cultural Differences

On a broader scale, these results parallel the robust findings documenting differences between East Asian and Western cultures (see Heine, 2001, 2003; Spencer-Rodgers et al., 2010 for reviews). TTEA inventory therefore begins to document a comprehensive set of ideological tenets that could be underlying those cultural differences. For example, future work could examine if differences on Restrictive Confucianism or any of its specific tenets might help explain the marked differences in face management (e.g., Ting-Toomey and Oetzel, 2001; Oetzel and Ting-Toomey, 2003), filial piety (e.g., Lee, 1995), and social anxiety (e.g., Heinrichs et al., 2006) observed between East Asian and Western samples. Similarly, future work on enculturation and acculturation could also use the TTEA inventory to clarify specific tenets that are more readily shaped by the dominant culture of the country in which one lives from the tenets that are more firmly acquired from the family interactions.

#### Internalizing Ideologies/Tenets Might Be Distinct From Religious/Spiritual Identities

Although it might be anticipated that religious identification/identity would be strongly linked to endorsing the tenets and beliefs of the corresponding religion (e.g., Templeton and Eccles, 2006), the current findings do not support that possibility. The composites of the TTEA inventory showed only low levels of correlation with dichotomous variables coding self-identified religious/spiritual affiliations. These findings are consistent with recent work highlighting that independent of religious identities, individuals can internalize the beliefs of religions to widely varying degrees and for different motives with correspondingly different outcomes (Ryan et al., 1993). The current results extend that previous work (focused primarily on Christianity), by highlighting the diversity and heterogeneity of internalized beliefs/tenets that exist even within populations of individuals identifying as Buddhist or Taoist. These results therefore underscore the critical utility of the TTEA inventory as a more direct measure of the internalization of the various tenets and beliefs examined. The correlational results would specifically suggest that a single item asking for self-reported spiritual/religious affiliations would likely not serve as an effective proxy to replace the direct assessment of the 18 tenets within the TTEA inventory, particularly if a researcher is trying to examine the influence of Buddhism, Taoism, or Confucianism on the lives of individuals or as a mechanism explaining cultural differences.

### Constraints on Generalizability

Using a large item pool given to four online samples representing five distinct cultural groups, the current study developed the TTEA inventory, a comprehensive and sophisticated framework to assess East Asian ideologies and the specific tenets from which they are formed. The TTEA inventory not only offers a sets of practical tools for research interested in understanding East Asians and their unique worldviews, but it also provides an empirically grounded method of raising one's cultural awareness to the unique perspectives of clients of East Asian descent. Despite these strengths, the results of the current study were limited by a number of factors. First, the study employed a cross-sectional design. Future work could examine these philosophical principles longitudinally and see whether they demonstrate developmental changes across major life transitions. Second, although the TTEA scales demonstrated consistently high levels of internal consistency across gender and cultural groups, future longitudinal studies should examine the test-retest correlations of the TTEA scales to more directly test their reliabilities. Future longitudinal studies would also help to clarify and quantify the prospective predictive validity of the TTEA subscales, thereby characterizing how the various tenets of Buddhism, Taoism, and Confucianism shape behavior and individual functioning over time. Third, the current study made use of self-report data, limiting the quality of our data to what individuals were willing and able to accurately report. Future work could build on this by using indirect methods to assess implicit views of the world and morality that could also shape behavior and well-being. Fourth, the data was collected entirely online, potentially introducing barriers to participation for subjects with lower levels of income and education and accessibility to technology. The sample was also predominantly female, raising potential concerns about the generalizability of the findings to males. Thankfully, the large size of the sample still offered 648 male respondents, and the analyses suggested that the TTEA subscales still functioned well within the male subgroups of each cultural group. Despite these promising findings, future studies could seek more diverse samples with higher proportions of males to ensure that the findings will generalize beyond the current sample. Fifth, extending the previous point, although the current study was unique in its efforts to develop and validate a new scale simultaneously across four languages and five cultural groups, the five cultural groups examined are not representative of all possible East Asian cultures, nor were they fully representative of the racial and ethnic diversity within the United States. Thus, future work on the TTEA inventory should examine its validity in other East Asian cultures (e.g., South Korea, Singapore, Hong Kong), in other racial/ethnic groups within the United States (e.g., African Americans/Black, Hispanic/Latinx, Native Americans), and across other continents (e.g., European cultures, African cultures). Finally, despite demonstrating clear discriminant validity from collectivism and dimensions of mindfulness, future work could further examine the discriminant validity of the TTEA inventory with a broader range of cultural constructs (e.g., contrasting the TTEA subscales from related constructs like the independent vs. interdependent self). Although these limitations open clear directions for future work, the current results suggest that the TTEA inventory offers cross-cultural researchers a powerful new tool for examining differences between Eastern and Western ideological orientations.

## Data Sharing Statement

The IRB materials, all four translations of the entire survey and study materials, and the SPSS and Mplus syntax used have been made available by the authors on the osf.io website.

## Data Availability Statement

The raw data supporting the conclusions of this article have been made available by request via online platform osf.io (https://osf.io/rvpg3/). Please contact the corresponding author to access the data.

## Ethics Statement

The studies involving human participants were reviewed and approved by Research Subjects Review Board at the University of Rochester. Written informed consent for participation was not required for this study in accordance with the national legislation and the institutional requirements.

## Author Contributions

Y-YL and DS originally conceptualized the project. Y-YL oversaw the translation and back-translation processes and took primary responsibility in recruiting the samples. Y-YL and RR implemented the survey on SurveyGizmo.com, cleaned and analyzed the data, and took the lead in writing the manuscript. All authors were involved in study design, obtaining necessary human subjects approval, contributed to the content of manuscript, agree with that content, and the statistical findings presented.

## Conflict of Interest

The authors declare that the research was conducted in the absence of any commercial or financial relationships that could be construed as a potential conflict of interest.
