# Supplementary material for: The Three Teachings of East Asia (TTEA) Inventory: Developing and Validating a Measure of the Interrelated Ideologies of Confucianism, Buddhism, and Taoism
Source: Front Psychol. 2021 Mar 1;12:626122. doi: 10.3389/fpsyg.2021.626122 (PMC7956942; doi:10.3389/fpsyg.2021.626122)
Supplement: Supplementary file 1 [file Table_1.docx]

**Supplemental Table S1. Items Drawn from Existing Scales and Created for the Current Study**

| **Construct / Subscale** | **Measure** | **Citation** | **Items** | **New Items** |  |
| --- | --- | --- | --- | --- | --- |
| **CONFUCIANISM** | | | | | |
| Interpersonal Harmony | East Asian Relational Norms Inventory | EARN_I (Park et al., 2011) | 5 | - |  |
| Interpersonal Harmony | East Asian Relational Norms | EARN (Park et al., 2009) | 1 | - |  |
| Propriety | East Asian Relational Norms Inventory | EARN_I (Park et al., 2011) | 5 | - |  |
| Relational Hierarchy | East Asian Relational Norms Inventory | EARN_I (Park et al., 2011) | 5 | - |  |
| Relational Hierarchy | East Asian Relational Norms | EARN (Park et al., 2009) | 3 | - |  |
| Self-cultivation | developed for study | N/A | - | 5 |  |
| Leading by example | developed for study | N/A | - | 5 |  |
| Heterogeneous life values | developed for study based loosely on items of the Confucian work values scale | CWVS (Lin, Ho, & Lin, 2013) | - | 6 |  |
| Well-defined roles | East Asian Relational Norms | EARN (Park et al., 2009) | 2 | - |  |
| Well-defined roles | Asian Values Scale | ASU (Kim, Atkinson, & Yang, 1999) | 2 | - |  |
| Well-defined roles/Conformity to Norms | Asian American Values Scale-Multidimensional | AAVS-M (Kim, Li, & Ng, 2005) | 3 | - |  |
| Well-defined roles | developed for study | N/A | - | 4 |  |
| Human heartedness | developed for study | N/A | - | 5 |  |
| SUM |  |  | **26** | **25** |  |
| **BUDDHISM** | | | | | |
| Impermanence | Buddhist Coping Measure | BCOPE (Phillips, Oemig, & Vonnegut, 2012) | 4 | 1 |  |
| Inter-being | Buddhist Coping Measure | BCOPE (Phillips, Oemig, & Vonnegut, 2012) | 4 | 1 |  |
| Not-self (Releasing attachment to the self) | Buddhist Coping Measure | BCOPE (Phillips, Oemig, & Vonnegut, 2012) | 4 | 1 |  |
| Punishing Karmic view | Buddhist Coping Measure | BCOPE (Phillips, Oemig, & Vonnegut, 2012) | 4 | 1 |  |
| Active Karmic view | Buddhist Coping Measure | BCOPE (Phillips, Oemig, & Vonnegut, 2012) | 4 | 2 |  |
| Meditation | Buddhist Coping Measure | BCOPE (Phillips, Oemig, & Vonnegut, 2012) | 5 | - |  |
| Mindfulness | Buddhist Coping Measure | BCOPE (Phillips, Oemig, & Vonnegut, 2012) | 2 | - |  |
| Present Moment Awareness | Multidimensional Psychological Flexibility Inventory | MPFI (Rolffs, Rogges, & Wilson, 2016) | 5 | - |  |
| SUM |  |  | **32** | **6** |  |
| **TAOISM** | | | | | |
| Embracing Contradiction | Dialectical Self Scale | DSS (Spencer-Rodgers et al, 2010) | 2 | 4 |  |
| Accepting Thing as They Are/ Zi-Ran | Daoist Thinking Style | DQ (Cott, 2012) | 3 | 4 |  |
| Non-Interference/ Wu Wei | Daoist Thinking Style | DQ (Cott, 2012) | 4 | 2 |  |
| Tranquility / Taoist Vision of Self | Daoist Thinking Style | DQ (Cott, 2012) | 4 | - |  |
| Cyclic Nature | developed for study | N/A | - | 10 |  |
| SUM |  |  | **13** | **20** |  |

**Supplemental Table S2.** *Cronbach Alpha Coefficients Demonstrating Generalizability of Internal Consistencies of the TTEA Subscales across Culture Groups Split by Gender*

| Ideology Composite | | Full Sample (n=2091) | | |  | Chinese (n=322) | | |  | Japanese (n=400) | | |  | Taiwanese (n=362) | | |  | | Asian Americans (n=319) | | |  | White Americans (n=688) | | | | |
| --- | --- | --- | --- | --- | --- | --- | --- | --- | --- | --- | --- | --- | --- | --- | --- | --- | --- | --- | --- | --- | --- | --- | --- | --- | --- | --- | --- |
|  | Tenet subscales | all | M | F |  | all | M | F |  | all | M | F |  | all | M | F |  | all | | M | F |  | all | M | F | |  |
| Number of Males/Females | |  | 658 | 1430 |  |  | 168 | 153 |  |  | 115 | 284 |  |  | 119 | 243 |  |  | | 128 | 190 |  |  | 128 | 560 | |  |
| Buddhism | |  |  |  |  |  |  |  |  |  |  |  |  |  |  |  |  |  | |  |  |  |  |  |  | |  |
| Full 16-item composite | | .93 | .94 | .93 |  | .91 | .92 | .90 |  | .93 | .94 | .93 |  | .91 | .93 | .90 |  | .94 | | .94 | .94 |  | .92 | .95 | .91 | |  |
| Shortened 10-item composite | | .89 | .91 | .88 |  | .86 | .87 | .83 |  | .89 | .90 | .89 |  | .86 | .89 | .84 |  | .90 | | .90 | .90 |  | .88 | .92 | .86 | |  |
|  | Not Self | .89 | .88 | .89 |  | .88 | .90 | .85 |  | .90 | .86 | .91 |  | .87 | .88 | .87 |  | .88 | | .88 | .88 |  | .89 | .86 | .89 | |  |
|  | Active Karmic View | .90 | .89 | .91 |  | .79 | .78 | .81 |  | .90 | .88 | .91 |  | .77 | .81 | .75 |  | .89 | | .88 | .90 |  | .92 | .92 | .91 | |  |
|  | Interconnectedness | .91 | .91 | .91 |  | .89 | .89 | .89 |  | .90 | .90 | .91 |  | .89 | .89 | .89 |  | .92 | | .91 | .92 |  | .91 | .93 | .91 | |  |
|  | Practicing Meditation | .95 | .96 | .95 |  | .95 | .95 | .95 |  | .94 | .95 | .94 |  | .97 | .98 | .96 |  | .96 | | .95 | .96 |  | .95 | .96 | .95 | |  |
|  | Punishing Karmic View | .90 | .88 | .91 |  | .84 | .80 | .87 |  | .91 | .89 | .92 |  | .83 | .82 | .84 |  | .85 | | .86 | .85 |  | .92 | .90 | .93 | |  |
| Taoism | |  |  |  |  |  |  |  |  |  |  |  |  |  |  |  |  |  | |  |  |  |  |  |  | |  |
| Full 18-item composite | | .92 | .93 | .92 |  | .91 | .92 | .90 |  | .91 | .90 | .92 |  | .93 | .94 | .92 |  | .92 | | .92 | .92 |  | .90 | .92 | .89 | |  |
| Shortened 10-item composite | | .86 | .87 | .85 |  | .83 | .85 | .80 |  | .85 | .84 | .85 |  | .87 | .89 | .85 |  | .86 | | .85 | .86 |  | .83 | .86 | .83 | |  |
|  | Embracing Contradiction | .83 | .83 | .83 |  | .86 | .85 | .87 |  | .81 | .79 | .82 |  | .87 | .84 | .88 |  | .83 | | .80 | .84 |  | .81 | .83 | .80 | |  |
|  | Noninterference | .88 | .84 | .89 |  | .81 | .74 | .87 |  | .87 | .82 | .89 |  | .90 | .90 | .90 |  | .88 | | .86 | .89 |  | .90 | .90 | .90 | |  |
|  | Zi-Ran | .86 | .86 | .85 |  | .85 | .85 | .84 |  | .84 | .83 | .84 |  | .85 | .86 | .84 |  | .84 | | .80 | .86 |  | .83 | .85 | .82 | |  |
|  | Cyclic Nature | .91 | .91 | .92 |  | .88 | .84 | .91 |  | .92 | .89 | .93 |  | .91 | .91 | .91 |  | .90 | | .89 | .90 |  | .91 | .92 | .91 | |  |
|  | Tranquility | .84 | .84 | .84 |  | .86 | .87 | .84 |  | .85 | .86 | .84 |  | .86 | .87 | .85 |  | .86 | | .84 | .87 |  | .82 | .78 | .82 | |  |
| Restrictive Confucianism | |  |  |  |  |  |  |  |  |  |  |  |  |  |  |  |  |  | |  |  |  |  |  |  | |  |
| Full 15-item composite | | .87 | .88 | .86 |  | .84 | .85 | .84 |  | .86 | .87 | .85 |  | .87 | .86 | .87 |  | .89 | | .88 | .89 |  | .86 | .89 | .85 | |  |
| Shortened10-item | | .83 | .84 | .83 |  | .81 | .81 | .80 |  | .81 | .81 | .81 |  | .81 | .80 | .82 |  | .85 | | .84 | .85 |  | .82 | .84 | .81 | |  |
|  | Propriety Pressure | .90 | .89 | .91 |  | .88 | .87 | .87 |  | .88 | .90 | .88 |  | .88 | .87 | .89 |  | .91 | | .88 | .92 |  | .90 | .86 | .90 | |  |
|  | Intrinsic Propriety | .75 | .73 | .76 |  | .70 | .63 | .76 |  | .84 | .82 | .84 |  | .74 | .71 | .75 |  | .70 | | .76 | .67 |  | .72 | .75 | .73 | |  |
|  | Relational Hierarchy | .85 | .86 | .84 |  | .82 | .81 | .82 |  | .88 | .89 | .88 |  | .87 | .89 | .86 |  | .84 | | .82 | .85 |  | .83 | .89 | .81 | |  |
|  | Interpersonal Harmony | .85 | .83 | .85 |  | .75 | .77 | .69 |  | .87 | .87 | .87 |  | .84 | .84 | .84 |  | .87 | | .84 | .89 |  | .86 | .86 | .86 | |  |
|  | Conforming to Social Norms | .91 | .88 | .91 |  | .86 | .82 | .90 |  | .86 | .90 | .85 |  | .88 | .77 | .92 |  | .91 | | .90 | .91 |  | .90 | .87 | .90 | |  |
| Empowering Confucianism | |  |  |  |  |  |  |  |  |  |  |  |  |  |  |  |  |  | |  |  |  |  |  |  | |  |
| Full 12-item composite | | .94 | .94 | .94 |  | .91 | .91 | .91 |  | .93 | .93 | .93 |  | .92 | .93 | .91 |  | .93 | | .93 | .92 |  | .92 | .94 | .91 | |  |
|  | Shortened 10-item | .89 | .89 | .89 |  | .85 | .86 | .85 |  | .87 | .87 | .87 |  | .87 | .88 | .86 |  | .87 | | .88 | .86 |  | .86 | .89 | .85 | |  |
|  | Self-Cultivation | .90 | .90 | .90 |  | .88 | .88 | .87 |  | .90 | .91 | .90 |  | .89 | .89 | .89 |  | .85 | | .86 | .84 |  | .86 | .88 | .85 | |  |
|  | Leading by Example | .89 | .89 | .89 |  | .85 | .86 | .85 |  | .87 | .87 | .87 |  | .85 | .86 | .85 |  | .89 | | .88 | .89 |  | .88 | .90 | .88 | |  |
|  | Human Heartedness | .88 | .88 | .88 |  | .85 | .85 | .86 |  | .88 | .88 | .88 |  | .85 | .84 | .85 |  | .88 | | .90 | .85 |  | .86 | .89 | .84 | |  |
| NOTE: all = Results across males and females, M = Results in males, F = Results in females. This table presents the unstandardized Cronbach alpha coefficients for each scale. | | | | | | | | | | | | | | | | | | | | | | | | | |  | |
